# Circular RNA circLMO7 acts as a microRNA-30a-3p sponge to promote gastric cancer progression via the WNT2/β-catenin pathway

**DOI:** 10.1186/s13046-020-01791-9

**Published:** 2021-01-05

**Authors:** Jiacheng Cao, Xing Zhang, Penghui Xu, Haixiao Wang, Sen Wang, Lu Zhang, Zheng Li, Li Xie, Guangli Sun, Yiwen Xia, Jialun Lv, Jing Yang, Zekuan Xu

**Affiliations:** 1 Department of General Surgery, The First Affiliated Hospital of Nanjing Medical University, Nanjing, 210029 Jiangsu Province China; 2 Collaborative Innovation Center For Cancer Personalized Medicine, Nanjing Medical University, Nanjing, 210029 Jiangsu Province China; 3 Department of General Surgery, The Affiliated Huaian No.1 People’s Hospital of Nanjing Medical University, Huaian, 223300 Jiangsu Province China

**Keywords:** Gastric cancer, circRNA, miRNA, WNT2, Glutaminolysis, HNRNPL

## Abstract

**Background:**

Gastric cancer (GC) is one of the most common malignant tumors worldwide. Currently, the overall survival rate of GC is still unsatisfactory despite progress in diagnosis and treatment. Therefore, studying the molecular mechanisms involved in GC is vital for diagnosis and treatment. CircRNAs, a type of noncoding RNA, have been proven to act as miRNA sponges that can widely regulate various cancers. By this mechanism, circRNA can regulate tumors at the genetic level by releasing miRNA from inhibiting its target genes. The WNT2/β-Catenin regulatory pathway is one of the canonical signaling pathways in tumors. It can not only promote the development of tumors but also provide energy for tumor growth through cell metabolism (such as glutamine metabolism).

**Methods:**

Through RNA sequencing, we found that hsa_circ_0008259 (circLMO7) was highly expressed in GC tissues. After verifying the circular characteristics of circLMO7, we determined the downstream miRNA (miR-30a-3p) of circLMO7 by RNA pull-down and luciferase reporter assays. We verified the effect of circLMO7 and miR-30a-3p on GC cells through a series of functional experiments, including colony formation, 5-ethynyl-2′-deoxyuridine and Transwell assays. Through Western blot and immunofluorescence analyses, we found that WNT2 was the downstream target gene of miR-30a-3p and further confirmed that the circLMO7-miR-30a-3p-WNT2 axis could promote the development of GC. In addition, measurement of related metabolites confirmed that this axis could also provide energy for the growth of GC cells through glutamine metabolism. We found that circLMO7 could promote the growth and metastasis of GC in vivo by the establishment of nude mouse models. Finally, we also demonstrated that HNRNPL could bind to the flanking introns of the circLMO7 exons to promote circLMO7 cyclization.

**Results:**

CircLMO7 acted as a miR-30a-3p sponge affecting the WNT2/β-Catenin pathway to promote the proliferation, migration and invasion of GC cells. Moreover, animal results also showed that circLMO7 could promote GC growth and metastasis in vivo. CircLMO7 could also affect the glutamine metabolism of GC cells through the WNT2/β-Catenin pathway to promote its malignant biological function. In addition, we proved that HNRNPL could promote the self-cyclization of circLMO7.

**Conclusions:**

CircLMO7 promotes the development of GC by releasing the inhibitory effect of miR-30a-3p on its target gene WNT2.

**Supplementary Information:**

The online version contains supplementary material available at 10.1186/s13046-020-01791-9.

## Background

Gastric cancer (GC) is one of the most common malignancies worldwide. As of 2018, the global incidence of GC ranks fifth among all cancers, while its mortality ranks third [1]. The 5-year survival rate of GC is not satisfactory. From 2005 to 2009, the 5-year survival rate of GC in China was only 31% [2]. The traditional diagnosis of GC is endoscopy and pathology [3]. However, the 5-year survival rate of GC patients is not high because the cancer has already metastasized at the time of diagnosis [4]. Therefore, further research on the molecular mechanisms involved in GC is extremely important for the early diagnosis and prognostic evaluation of tumors.

Circular RNA (circRNA) is a class of noncoding RNA molecules connected end-to-end in a covalently closed loop structure [5]. Currently, circRNAs are known to be widely present in the cytoplasm of eukaryotic cells and are highly conserved during species evolution [6, 7]. The formation of circRNA is closely related to the reverse splicing of its precursor mRNA (pre-mRNA) [8, 9]. The vast majority of circRNAs are generated by exon circularization, while some are circularized by introns, and few are composed of exons and introns together [10]. Compared with traditional linear RNA, circRNA does not have a 5′ cap or a 3′ poly(A) tail structure. This structural feature makes it more stable and difficult to degrade by exonuclease [11]. In recent years, circRNAs have been reported to play an increasingly important role in the study of new tumor biomarkers due to these superior characteristics [10]. The role of circRNAs in tumorigenesis and development is mainly summarized as follows. CircRNA can be used as competitive endogenous RNA (ceRNA) to regulate microRNA (miRNA) [12–16]. CircRNA can also bind to RNA-binding protein (RBP) to affect the biological malignancy of tumors [5]. Interestingly, some RBPs can also bind to the flanking introns of related pre-mRNA exons and affect the formation of circRNAs [17]. Some circRNAs can act as transcription regulators [18]. In addition, very few circRNAs can directly encode polypeptides [19].

With in-depth studies of the mechanism of tumorigenesis, it has been reported that glutamine metabolism is one of the most important metabolic modes in cancer and that some circRNAs can promote the development of cancer through it [20]. For example, circHECTD1 and circHMGCS1 can promote the development of GC and hepatoblastoma by glutamine decomposition, respectively [21, 22]. With the catalysis of glutaminase (GLS), glutamine (GLN) can be converted into glutamate (GLU), which is then converted into α-ketoglutarate (α-KG) by glutamate dehydrogenase (GDH) to participate in the tricarboxylic acid cycle (TCA cycle) and provide energy for tumor growth [20, 23]. In addition, glutamate, together with cysteine ​​and glycine, can produce glutathione (GSH), which neutralizes peroxy radicals to maintain reactive oxygen species (ROS) stress balance and reduce tumor cell damage caused by oxidative stress [24, 25].

In this study, we confirmed that circLMO7 can absorb miR-30a-3p to affect the WNT2/β-Catenin pathway to promote the development of GC. Moreover, circLMO7 can also promote the glutamine metabolism of GC cells through the WNT2/β-Catenin pathway to provide energy for tumor growth. In addition, HNRNPL can bind to the flanking introns of the circLMO7 exon, thereby promoting the self-cyclization of circLMO7. In short, the study of circLMO7 as a new biomarker and a potential target for GC treatment is very promising.

## Supplementary materials and methods

All the materials and methods are included in the Supplementary Materials and Methods section.

## Results

### Differentially expressed circRNAs in GC were detected by NGS

**Fig. 1:**
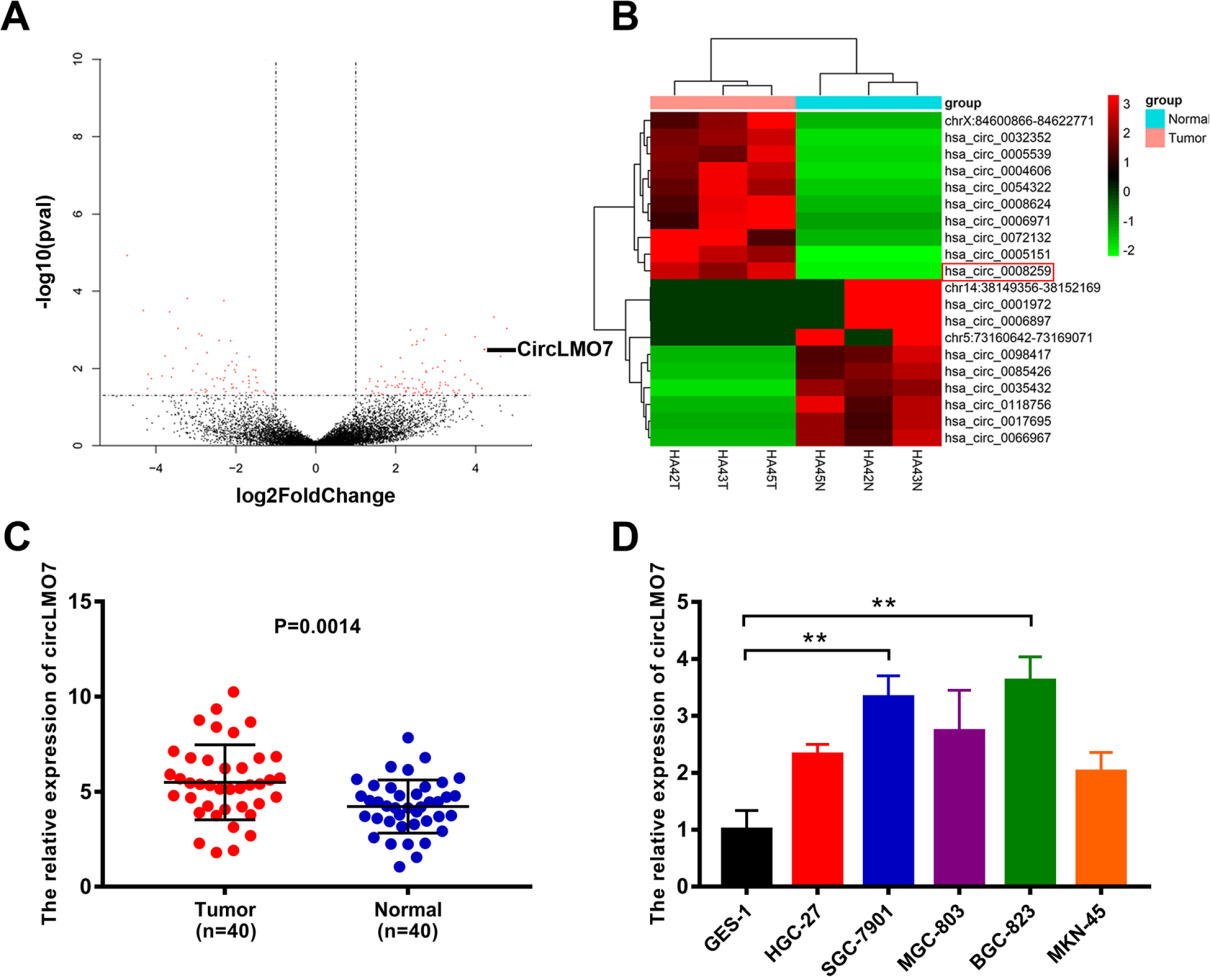
Differentially expressed circRNAs in GC were detected by NGS. **a**, **b**. Volcano map and cluster heat map showing the differences in the expression of circRNA in GC tissues and adjacent normal tissues. **c**. qRT-PCR showed that the expression of circLMO7 in GC tissues was significantly higher than that in adjacent normal tissues. **d**. Different expression levels of circLMO7 in cell lines. All data are presented as the mean ± SD. * *P* < 0.05, ** *P* < 0.01, *** *P* < 0.001

We performed NGS in the absence of ribosomal RNA on GC tissues and adjacent normal tissues from three people. Sequencing results showed that a total of 450 circRNAs were differentially expressed, of which 314 circRNAs were upregulated and 136 circRNAs were downregulated. Based on the volcano map and cluster heat map of the sequencing data, a significantly upregulated circRNA (chr13: 76287318–76,335,174, hsa_circ_0008259, referred to as circLMO7) aroused our interest (Fig. 1a, b). To further verify the expression of circLMO7 in GC tissues, we performed qRT-PCR on 40 paired tissues. The results showed that the expression of circLMO7 in GC tissues was significantly higher than that in adjacent normal tissues (Fig. 1c). This result was consistent with the NGS analysis. Next, we verified the expression of circLMO7 in cell lines, and the results suggested that the expression of circLMO7 in HGC-27, SGC-7901, MGC-803, BGC-823 and MKN-45 cells was significantly increased relative to that in GES-1 cells. Among these cell line, BGC-823 cells had the highest expression, followed by SGC-7901 cells (Fig. 1d). Therefore, we selected BGC-823 and SGC-7901 cells to study the regulatory pathway of circLMO7. Next, by Sanger sequencing, we found that circLMO7 originated from the LMO7 gene on human chromosome 13. This circRNA was formed by exons 3, 4, and 5 (76287318–76,335,174) of the gene from end to end, and the loop site of circLMO7 is indicated in the sequencing results (Supplementary Fig. 1A). RNA fluorescence in situ hybridization (FISH) further confirmed the presence of circLMO7 (Supplementary Fig. 1B). To verify the circularity of circLMO7, we treated total RNA of GC cells with RNase R and then performed qRT-PCR with divergent and convergent primers. The results showed that circLMO7 was significantly less susceptible to RNase R digestion than linear LMO7 (Supplementary Fig. 1C). Next, we performed agarose gel electrophoresis on the qRT-PCR products. The results showed that the qRT-PCR products without RNase R treatment contained amplified sequences corresponding to circLMO7 and linear LMO7. However, the qRT-PCR products with RNase R treatment only contained the amplified sequence corresponding to circLMO7. In addition, the genomic DNA (gDNA) qRT-PCR products only contained the amplified sequence corresponding to linear LMO7, which was determined by the positional characteristics of circLMO7 and linear LMO7 (Supplementary Fig. 1D). Moreover, the actinomycin D inhibition test also proved that circLMO7 was more stable than linear LMO7 (Supplementary Fig. 1E). In summary, we found that circLMO7 was significantly more stable than linear LMO7. The expression of circLMO7 could also affect the clinicopathological characteristics of GC. We divided the 40 patients into a high expression group and a low expression group according to the median expression of circLMO7 in GC tissues. The analysis showed that the expression of circLMO7 was closely related to the T grade and stage of GC. However, circLMO7 expression was not associated with the gender, lymphatic invasion, size, tumor site or Lauren classification of GC (Table 1). Collectively, the results showed that circLMO7 has a stable circular structure and certain effects on the clinicopathological characteristics of GC.

**Table 1:** The influence on clinicopathological characteristics of gastric cancer

Parameters	Group	CircLMO7 expression			
		Cases	Low	High	*P*-value
Gender	Male	21	10	11	0.752
	Female	19	10	9	
T grade	T1 + T2	7	7	0	0.008**
	T3 + T4	33	13	20	
Lymphatic invasion	Negative(N0)	5	4	1	0.342
	Positive(N1-N3)	35	16	19	
Stage	I-II	9	8	1	0.020*
	III-IV	31	12	19	
Size(cm)	< 3	10	5	5	1.000
	≥3	30	15	15	
Tumor site	Cardiac	4	3	1	0.605
	Non-cardiac	36	17	19	
Lauren classification	Intestinal type carcinoma	9	5	4	1.000
	Non-Intestinal type carcinoma	31	15	16	

*p* < 0.05 represents statistical significance (Chi-square test)

### CircLMO7 acts as a sponge of miR-30a-3p

**Fig. 2:**
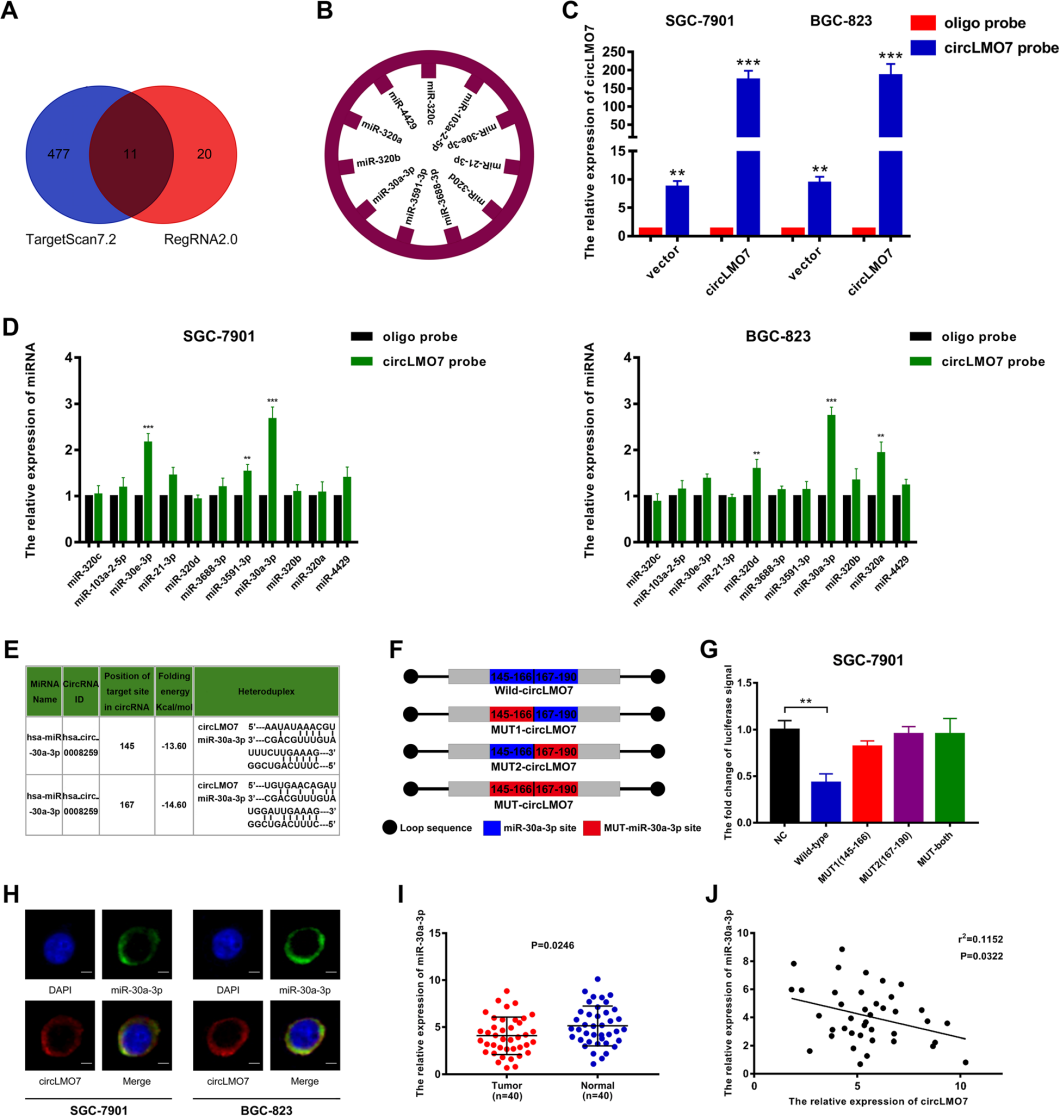
CircLMO7 acts as a sponge of miR-30a-3p. **a**. Venn diagram showing the target miRNAs of circLMO7 predicted by TargetScan7.2 and RegRNA2.0. **b**. Target miRNAs overlapping between TargetScan7.2 and RegRNA2.0. **c**. Pull-down efficiency of the circLMO7 probe. **d** After pull-down experiments, we found that the binding efficiency between circLMO7 and miR-30a-3p was the highest. **e**. Sequence diagram of the binding sites between circLMO7 and miR-30a-3p. **f**. Map of the mutations of the binding sites between circLMO7 and miR-30a-3p. **g**. A luciferase reporter assay demonstrated that circLMO7 and miR-30a-3p bound to each other. **h**. RNA fluorescence in situ hybridization confirmed the binding relationship between circLMO7 and miR-30a-3p; scale bar = 10 μm. i. qRT-PCR showed that the expression of miR-30a-3p in GC tissues was significantly lower than that in adjacent normal tissues. **j**. qRT-PCR showed that circLMO7 and miR-30a-3p levels were negatively correlated. All data are presented as the mean ± SD. * *P* < 0.05, ** *P* < 0.01, *** *P* < 0.001

Next, we explored the sponge function of circLMO7 and miRNA. By using TargetScan7.2 (http://www.targetscan.org/vert_72/) and RegRNA2.0 (http://regrna2.mbc.nctu.edu.tw/detection.html) to predict the target miRNA of circLMO7, we found that circLMO7 can bind to 488 and 31 potential miRNAs based on TargetScan7.2 and RegRNA2.0, respectively. After the results of the two databases were combined, 11 overlapping miRNAs were identified (Fig. 2a), namely, miR-320c, miR-103a-2-5p, miR-30e-3p, miR-21-3p, miR-320d, miR-3688-3p, miR-3591-3p, miR-30a-3p, miR-320b, miR-320a, and miR-4429 (Fig. 2b). Next, we verified the ability of circLMO7 to bind to these 11 miRNAs by RNA pull-down experiments. First, we measured the pull-down efficiency of the circLMO7 probe in SGC-7901 and BGC-823 cells by qRT-PCR. The results showed that the efficiency of the circLMO7 probe was significantly higher than that of the oligo probe (Fig. 2c). We then performed RNA pull-down and used qRT-PCR to detect the expression of the 11 miRNAs in the pull-down products. We found that miR-30a-3p had the highest expression level in both SGC-7901 and BGC-823 cells, which suggested the highest binding efficiency (Fig. 2d). In addition, we identified two binding sites between circLMO7 and miR-30a-3p according to RegRNA2.0 (Fig. 2e). Next, the luciferase reporter assay was used to further verify the predicted binding. We constructed a circLMO7 luciferase reporter vector and its mutants and then carried out the experiment (Fig. 2f). The results demonstrated that cotransfection of wild-type circLMO7 with miR-30a-3p mimics caused a significant decrease in luciferase activity, while cotransfecting mutant circLMO7 (in which one of the binding sites or both binding sites were mutated) and miR-30a-3p mimics did not have the same effect (Fig. 2g), indicating the binding between circLMO7 and miR-30a-3p. In addition, RNA fluorescence in situ hybridization confirmed the colocalization of circLMO7 and miR-30a-3p in the cytoplasm (Fig. 2h). To further explore whether there was a quantitative relationship between circLMO7 and miR-30a-3p, we measured the expression of miR-30a-3p in the 40 paired tissues by qRT-PCR (Fig. 2i) and analyzed its correlation with circLMO7. The results showed that the expression levels of circLMO7 and miR-30a-3p were negatively correlated in GC tissues (Fig. 2j). In summary, circLMO7 can bind to miR-30a-3p as a sponge and is negatively correlated with miR-30a-3p.

### Overexpression of circLMO7 or knockdown of miR-30a-3p promotes the proliferation, migration and invasion of GC cells

**Fig. 3:**
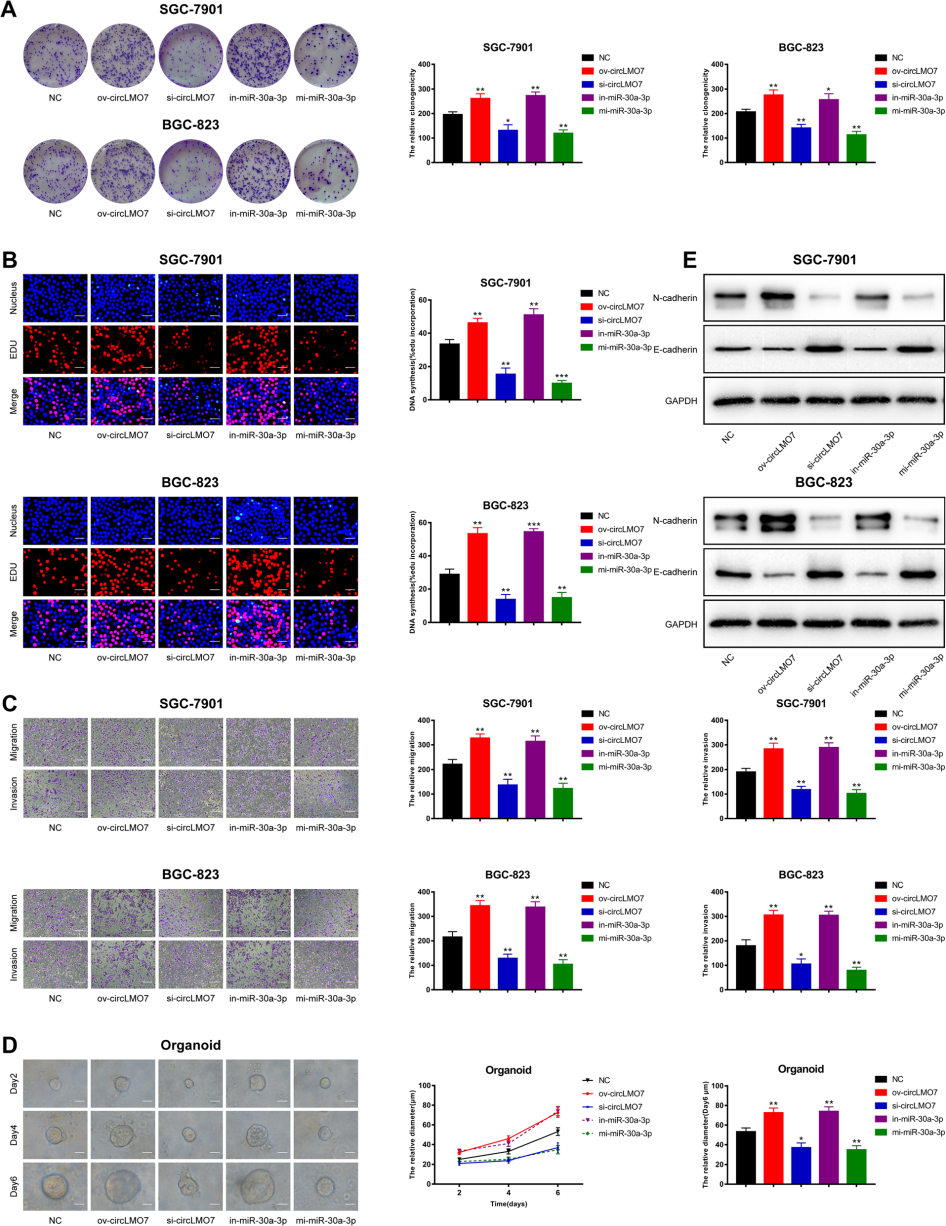
Overexpression of circLMO7 or knockdown of miR-30a-3p promotes the proliferation, migration and invasion of GC cells. **a**, **b**. Colony formation and EdU assays showed that overexpression of circLMO7 or knockdown of miR-30a-3p promoted the proliferation of GC cells, while knockdown of circLMO7 or overexpression of miR-30a-3p inhibited this process; EdU scale bar = 25 μm. **c**. Transwell assays showed that overexpression of circLMO7 or knockdown of miR-30a-3p promoted the migration and invasion of GC cells, while knockdown of circLMO7 or overexpression of miR-30a-3p had the opposite effects; scale bar = 100 μm. **d**. Overexpression of circLMO7 or knockdown of miR-30a-3p promoted the growth of human GC organoids, while the growth of human GC organoids was inhibited after circLMO7 had been knocked down or miR-30a-3p had been overexpressed; scale bar = 20 μm. **e**. Overexpression of circLMO7 or knockdown of miR-30a-3p promoted the migration and invasion of GC cells through the EMT pathway, while knockdown of circLMO7 or overexpression of miR-30a-3p showed the opposite results. All data are presented as the mean ± SD. * *P* < 0.05, ** *P* < 0.01, *** *P* < 0.001

To further explore the underlying functions of circLMO7 and miR-30a-3p in the development of GC, we constructed small interfering RNAs specific to circLMO7 (si-circLMO7), a plasmid overexpressing circLMO7 (ov-circLMO7), miR-30a-3p mimics (mi-miR-30a-3p), and a miR-30a-3p inhibitor (in-miR-30a-3p). We transfected these oligonucleotide sequences into SGC-7901 and BGC-823 cells and first verified that si-circLMO7 and ov-circLMO7 could silence and amplify circLMO7, respectively, before performing functional experiments. Transfection of these reagents only affected circLMO7 and not the linear form (Supplementary Fig. 2A, B). In addition, the effects of mi-miR-30a-3p and in-miR-30a-3p were also confirmed (Supplementary Fig. 2C). Next, we used colony formation, 5-ethynyl-2′-deoxyuridine (EdU) and Transwell assays to determine the proliferation, migration and invasion ability of GC cells, respectively. The assay results showed that overexpression of circLMO7 or knockdown of miR-30a-3p could promote the proliferation, migration and invasion of GC cells in vitro. In contrast, knocking down circLMO7 or overexpressing miR-30a-3p showed the opposite results (Fig. 3a-c). In recent years, human organoids have become a hot topic in tumor research because they simulate the real human environment [26–28]. We found that overexpressing circLMO7 or knocking down miR-30a-3p could promote the growth of human GC organoids. Accordingly, knocking down circLMO7 or overexpressing miR-30a-3p showed the opposite results (Fig. 3d). Epithelial-mesenchymal transition (EMT) refers to the process by which epithelial cells transform into mesenchymal cells, which promotes the migration and invasion of cancer cells [29–31]. Western blotting confirmed that when overexpressing circLMO7 or knocking down miR-30a-3p, the mesenchymal cell marker N-cadherin was upregulated and the epithelial cell marker E-cadherin was downregulated (Fig. 3e), while knocking down circLMO7 or overexpressing miR-30a-3p showed the opposite results. This indicated that overexpression of circLMO7 or knockdown of miR-30a-3p can promote the migration and invasion of GC cells by promoting the EMT process.

### CircLMO7 promotes the expression of WNT2 and circLMO7 promotes the development of GC through the circLMO7-miR-30a-3p-WNT2 axis

**Fig. 4:**
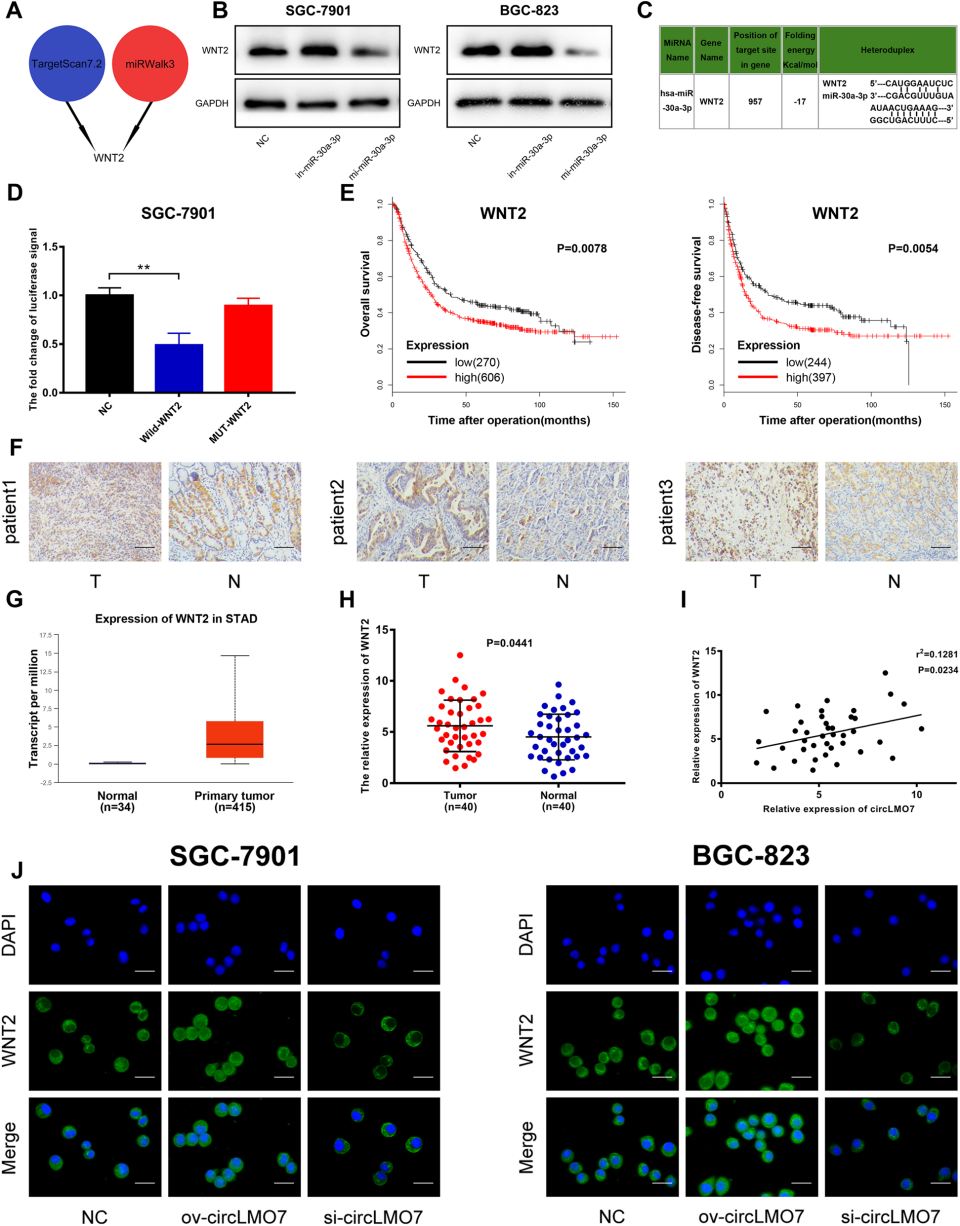
CircLMO7 promotes the expression of WNT2. **a**. Pattern diagram showing the downstream target gene of miR-30a-3p predicted by TargetScan7.2 and miRWalk3. **b**. Western blotting showed that the expression levels of miR-30a-3p and WNT2 were negatively correlated. **c**. Sequence map of the binding site between miR-30a-3p and WNT2. **d**. A luciferase reporter assay demonstrated that miR-30a-3p and WNT2 bind to each other. **e**. The KmPlot database showed that patients with lower WNT2 had better overall survival rates and disease-free survival rates than patients with higher WNT2. **f**. Immunohistochemistry showed that WNT2 was highly expressed in GC tissues; scale bar = 100 μm. **g**. The TCGA database showed that the expression of WNT2 in GC tissues was significantly higher than that in normal tissues. **h**. qRT-PCR showed that WNT2 was highly expressed in GC tissues. **i**. qRT-PCR showed that the expression levels of circLMO7 and WNT2 were positively correlated. **j**. Overexpression of circLMO7 promoted the expression of WNT2, while knockdown of circLMO7 inhibited the expression of WNT2; scale bar = 50 μm. All data are presented as the mean ± SD * *P* < 0.05, ** *P* < 0.01, *** *P* < 0.001

In the classical ceRNA mechanism, circRNA can relieve the inhibitory effect of miRNA on its target gene, thereby increasing the gene expression level. Therefore, we investigated the downstream target genes of miR-30a-3p. With the help of TargetScan7.2 (http://www.targetscan.org/vert_72/) and miRWalk3 (http://mirwalk.umm.uni-heidelberg.de/), we discovered the potential target gene WNT2 (Fig. 4a). WNT2 is an evolutionarily conserved secreted glycoprotein. It has been reported that WNT2 can promote the progression of GC [32–34]. Therefore, we hypothesized that WNT2 might be related to the circLMO7-miR-30a-3p axis and play a promoting role in tumor development. To validate the above hypothesis, we first verified the relationship between miR-30a-3p and WNT2 expression by Western blotting. The expression of WNT2 increased significantly after inhibition of miR-30a-3p. When miR-30a-3p was overexpressed, the opposite result was obtained (Fig. 4b). Next, we searched for the binding sites between miR-30a-3p and WNT2 (Fig. 4c) in TargetScan7.2 and verified the predicted sites with the luciferase reporter assay. The results showed that cotransfection of wild-type WNT2 and miR-30a-3p mimics caused a decrease in luciferase activity, while cotransfection of mutant WNT2 and miR-30a-3p mimics did not (Fig. 4d). Based on the above results, we confirmed that miR-30a-3p can bind to WNT2 and negatively regulate WNT2. Next, we found that WNT2 was highly expressed in GC tissues by immunohistochemical analysis and the TCGA database (https://www.cancer.gov/about-nci/organization/ccg/research/structural-genomics/tcga) (Fig. 4f, g). Moreover, the KmPlot database (http://kmplot.com/analysis/index.php?p=service) also showed that a higher WNT2 expression level was positively correlated with worse patient prognosis (Fig. 4e). The qRT-PCR data obtained for WNT2 in the 40 patients were consistent with the previous result (Fig. 4h). Next, we performed a linear correlation analysis between the expression levels of WNT2 and circLMO7, which indicated that they were positively correlated (Fig. 4i). Additionally, we confirmed the relationship between circLMO7 and WNT2 expression through immunofluorescence analysis (Fig. 4j). Here, we hypothesized that circLMO7 sponges miR-30a-3p to regulate WNT2, eventually promoting the development of GC. To confirm this hypothesis, we conducted rescue experiments. The results of the colony formation and EdU assays showed that the inhibitory effect of si-circLMO7 on GC cell proliferation was rescued after cotransfection with in-miR-30a-3p (Supplementary Fig. 3A, B). Moreover, the human GC organoids experiment showed the same results (Supplementary Fig. 3E). In addition, based on scratch assay, Transwell assay and Western blotting, we found that the inhibitory effect of si-circLMO7 on GC cell migration and invasion could be rescued after cotransfection with in-miR-30a-3p (Supplementary Fig. 3C, D, F). Next, we verified the expression levels of the target gene WNT2 and its downstream proteins, such as β-catenin, phosphorylated β-catenin (p-β-catenin) and matrix metalloproteinase-9 (MMP9). The results showed that the expression levels of these proteins were decreased when si-circLMO7 was transfected, but they were rescued after cotransfection with in-miR-30a-3p (Supplementary Fig. 3F). Therefore, we validated that si-circLMO7 inhibited GC cell proliferation, migration and invasion accompanied by decreased expression levels of WNT2 and its downstream proteins. The results were rescued when the cells were cotransfected with in-miR-30a-3p. In summary, circLMO7 can promote the development of GC through the circLMO7-miR-30a-3p-WNT2 axis.

### CircLMO7 can promote the growth and metastasis of GC in vivo

**Fig. 5:**
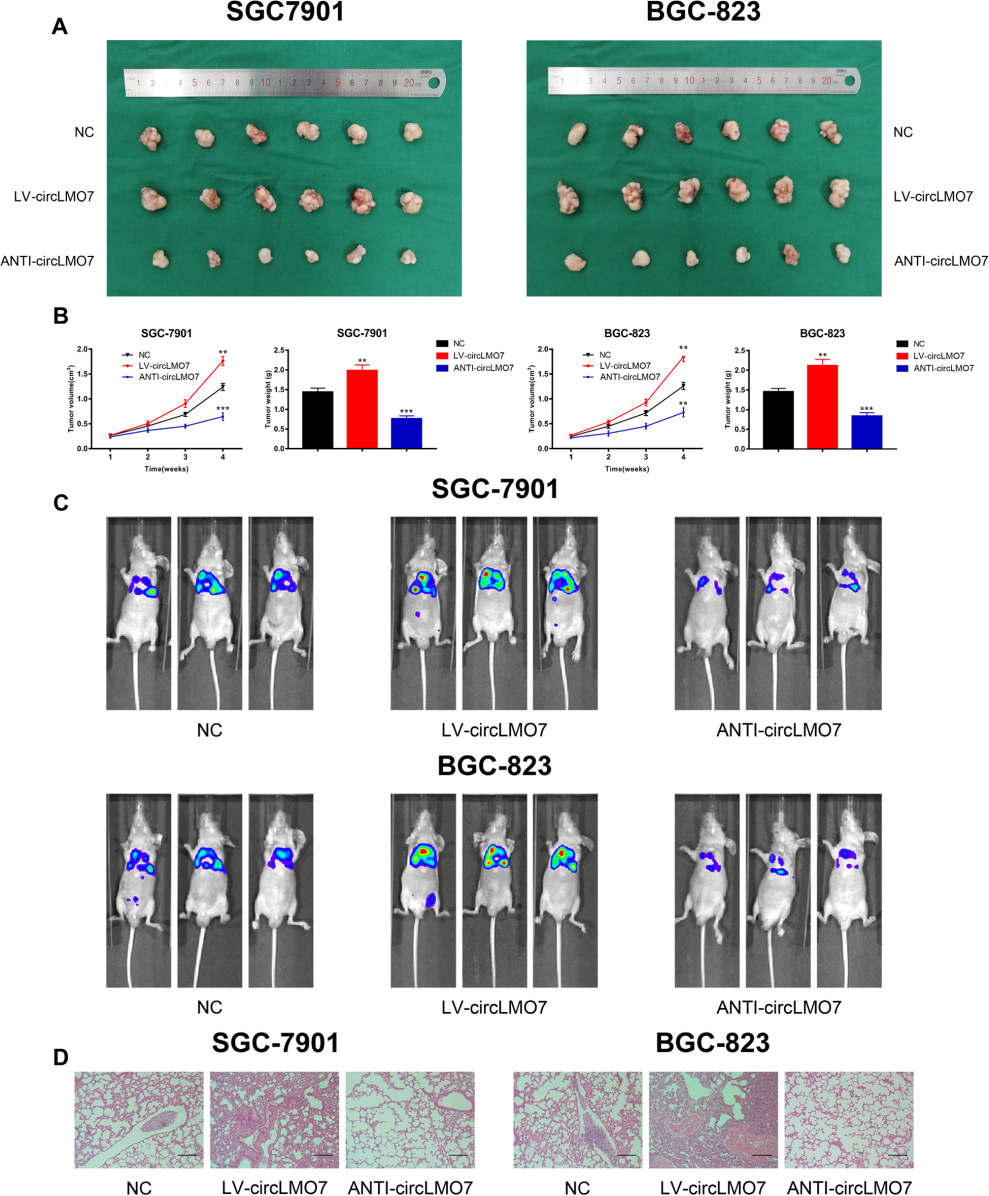
CircLMO7 can promote the growth and metastasis of GC in vivo. **a**. Overexpression of circLMO7 promoted the growth of xenograft tumors, while knockdown of circLMO7 inhibited tumor growth. **b**. Measurement of xenograft tumor volume and weight. **c**. Overexpression of circLMO7 promoted lung metastasis, while knockdown of circLMO7 inhibited lung metastasis. **d**. Hematoxylin-eosin staining showed the size of lung metastatic tissues; scale bar = 200 μm. All data are presented as the mean ± SD. * *P* < 0.05, ** *P* < 0.01, *** *P* < 0.001

Next, we further validated the cancer-promoting role of circLMO7 in GC by constructing a series of models in vivo. We randomly divided 36 BALB/c nude mice into three groups to construct xenograft tumor models. All nude mice were sacrificed after 4 weeks, and the weight and volume of xenograft tumors were measured (V = length×width^2^ × 0.5). The results showed that overexpression of circLMO7 significantly promoted the growth of xenograft tumors, while knocking down circLMO7 suppressed tumor growth (Fig. 5a). The weight and volume of xenograft tumors reflected this obvious difference (Fig. 5b). In addition, to verify the effect of circLMO7 on GC metastasis, we constructed lung metastasis models in 18 BALB/c nude mice. Four weeks after injecting 1 × 10^6^ cells into the tail vein of each mouse, the metastasis of lungs in nude mice was detected by IVIS. The results showed that overexpression of circLMO7 significantly promoted lung metastasis in nude mice, while knocking down circLMO7 reversed this effect (Fig. 5c). In addition, hematoxylin-eosin staining of the lung metastasis tissues suggested that the size of the tumor tissue was consistent with the above results (Fig. 5d). Overall, circLMO7 can promote the growth and metastasis of GC in vivo.

### CircLMO7 promotes GC malignant biological functions through glutamine metabolism

**Fig. 6:**
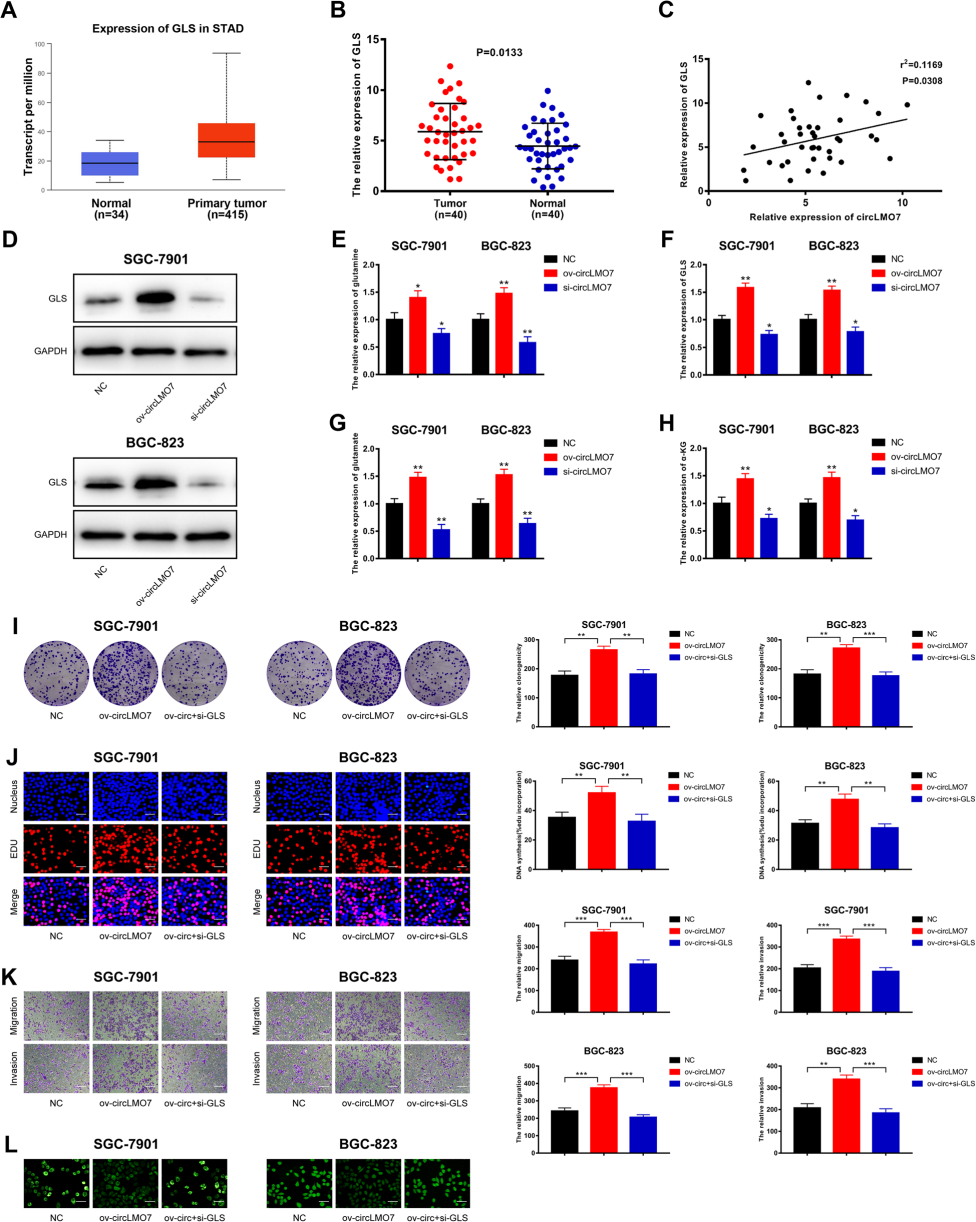
CircLMO7 promotes GC malignant biological functions through glutamine metabolism. **a**. TCGA database analysis showed that GLS was highly expressed in GC tissues. **b**. qRT-PCR verified that GLS was highly expressed in GC tissues. **c**, **d**. qRT-PCR and Western blot analysis showed that circLMO7 and GLS were positively related. **e**-**h**. Overexpressing circLMO7 increased the expression levels of GLN, GLS, GLU and α-KG. Knocking down circLMO7 blocked this phenomenon. **i**, **j**. Colony formation and EdU assays showed that the promoting effect of ov-circLMO7 on GC cell proliferation was rescued after cotransfection with si-GLS; EdU scale bar = 25 μm. **k**. Transwell assays showed that si-GLS rescued the promoting effect of ov-circLMO7 on GC cell migration and invasion; scale bar = 100 μm. **l**. The active oxygen assay showed that the inhibitory effect of ov-circLMO7 on the production of reactive oxygen species in GC cells was rescued after cotransfection with si-GLS; scale bar = 50 μm. All data are presented as the mean ± SD. * *P* < 0.05, ** *P* < 0.01, *** *P* < 0.001

Glutamine metabolism is one of the important metabolic modes of tumors. It has been reported that the WNT/β-catenin pathway can promote the malignant biological functions of tumors through glutamine metabolism [35, 36]. In the above section, we found that the circLMO7-miR-30a-3p-WNT2 axis can promote the development of GC. Therefore, we next wanted to explore whether this axis could also regulate glutamine metabolism to participate in GC development. Through circRNA-miRNA-mRNA pathway analysis, we found that the circLMO7-miR-30a-3p-WNT2 axis was closely related to glutamine metabolism (Supplementary Fig. 4). With the help of TCGA database, we found that GLS, a key enzyme for glutamine metabolism, was highly expressed in GC tissues (Fig. 6a). The qRT-PCR data on GLS expression in 40 patients was consistent with the TCGA database findings (Fig. 6b). Linear correlation analysis and Western blotting showed that circLMO7 was positively correlated with GLS (Fig. 6c, d). These results suggested that circLMO7 can regulate the expression level of GLS. Then, we found that overexpression of circLMO7 maintained the glutamine metabolism pathway in a highly active state by promoting the raw material GLN and the key enzyme GLS, as well as the expression of the intermediate metabolites GLU and α-KG. However, when circLMO7 was knocked down, the activity of the glutamine metabolism pathway was blocked (Fig. 6e-h). Next, colony formation and EdU assays showed that si-GLS rescued the promoting effect of ov-circLMO7 on GC cell proliferation (Fig. 6i, j). Transwell assays showed that the promoting effect of ov-circLMO7 on GC cell migration and invasion was also rescued after cotransfection with si-GLS (Fig. 6k). In addition, we found that overexpression of circLMO7 could inhibit the production of reactive oxygen species (ROS), but si-GLS rescued this effect as well (Fig. 6l). In summary, circLMO7 can promote the malignant biological functions of GC through glutamine metabolism.

### HNRNPL promotes the production of circLMO7 in GC

**Fig. 7:**
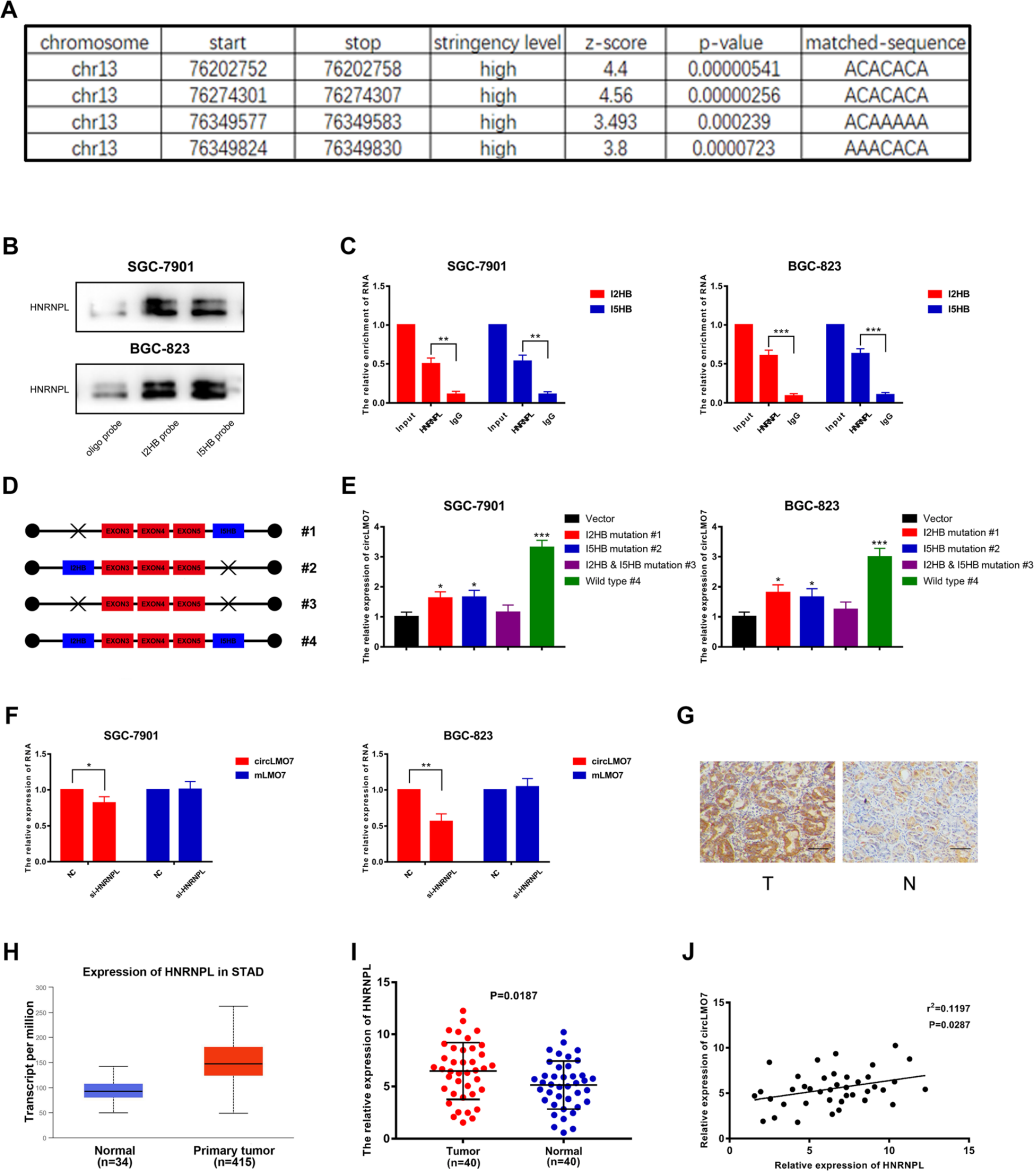
HNRNPL promotes the production of circLMO7 in GC. **a**. RBPmap showed the binding sites between the flanking introns of circLMO7 exons and HNRNPL. **b**, **c**. RNA pull-down and RNA immunoprecipitation assays showed that HNRNPL can bind to the flanking introns of circLMO7 exons. **d**, **e**. qRT-PCR showed that the wild-type plasmid could significantly increase the expression of circLMO7, while the mutant plasmids could not. **f**. qRT-PCR showed the expression levels of circLMO7 and pre-mLMO7 after knocking down HNRNPL. **g**. Immunohistochemistry showed that HNRNPL was highly expressed in GC tissues; scale bar = 100 μm. **h**. The TCGA database showed that the expression of HNRNPL in GC tissues was significantly higher than that in normal tissues. **i**, **j**. qRT-PCR showed that HNRNPL was highly expressed in GC tissues and was positively correlated with circLMO7. All data are presented as the mean ± SD. * *P* < 0.05, ** *P* < 0.01, *** *P* < 0.001

Most circRNAs are circularized by exons, and this process is affected by many factors. It has been reported that some RBPs can interact with the flanking introns of exons to induce exon circularization, which promotes the production of corresponding circRNAs [37, 38]. Such RBPs are HNRNPL, QKI, E2F1, EIF4A3, etc. [39–41]. This prompted us to investigate the formation of circLMO7. We investigated the above RBPs through RBPmap analysis (http://rbpmap.technion.ac.il/index.html) and found that HNRNPL had the highest binding efficiency with the flanking introns of the circLMO7 exons. Heterogeneous ribonucleoprotein L (HNRNPL) is a common RBP and has been reported to bind the specific flanking introns of exons to enhance the production of circRNA [39]. From the RBPmap analysis, we found that the flanking introns of circLMO7 exons had 4 binding sites with HNRNPL (Fig. 7a). Two of them were located in the second intron, which were named I2HB (intron 2 HNRNPL binding sequence). The other two were located in the fifth intron and were named I5HB (intron 5 HNRNPL binding sequence). Next, we verified the binding relationship between HNRNPL and the flanking introns by RNA pull-down and RNA immunoprecipitation assays (Fig. 7b, c). After that, we constructed a mutant plasmid and wild-type plasmid to verify the correlation between circLMO7 and I2HB & I5HB (Fig. 7d). qRT-PCR showed that only the wild-type plasmid could significantly upregulate the expression of circLMO7, while the mutant plasmids (I2HB/I5HB was mutated or both were mutated) did not have an effect (Fig. 7e). The results indicated that both I2HB and I5HB were indispensable for the production of circLMO7. Next, we investigated whether HNRNPL can regulate the production of circLMO7 at the posttranscriptional level. We found that when HNRNPL was knocked down, the expression of circLMO7 was significantly reduced, while the expression of pre-mLMO7 was not significantly changed (Fig. 7f). Combined with the results mentioned above, it was clear that HNRNPL affected the production of circLMO7 by interacting with its binding motif. In addition, we found that HNRNPL was highly expressed in GC tissues by immunohistochemistry (Fig. 7g), which was consistent with the TCGA database results (Fig. 7h). Moreover, qRT-PCR verified that HNRNPL was highly expressed in GC tissues (Fig. 7i), and the linear correlation analysis suggested that the expression levels of HNRNPL and circLMO7 were positively correlated (Fig. 7j). In summary, HNRNPL can bind to flanking introns of circLMO7 exons to promote its production.

## Discussion

In this study, we used NGS to identify a potential target circRNA (circLMO7) that is significantly upregulated in GC tissues. We verified the existence and circular characteristics of circLMO7 by Sanger sequencing and predicted its downstream miRNA (miR-30a-3p) through bioinformatics analysis. Through RNA pull-down and luciferase reporter assays, we found that circLMO7 and miR-30a-3p could bind to each other. Functional assays showed that circLMO7 could promote the proliferation, migration and invasion of GC cells. However, miR-30a-3p inhibited the above cell processes. Next, WNT2, a secreted glycoprotein, was predicted to be a potential target gene of miR-30a-3p by bioinformatics analysis. It has been reported that WNT2 can promote the development of tumors [42–44], and the WNT2/β-Catenin signaling pathway is related to the malignant biological functions of GC [45–47]. This protein drew our attention. We confirmed that miR-30a-3p could bind to WNT2 and found that circLMO7 promoted the development of GC through the circLMO7-miR-30a-3p-WNT2 axis. We also found that circLMO7 promoted GC growth and metastasis in vivo through animal experiments. Next, we conducted experiments to verify that circLMO7 could promote the malignant biological functions of GC by glutamine metabolism. Finally, we found that the RNA-binding protein HNRNPL can bind to the flanking introns of circLMO7 exon to promote its cyclization.

As far as the current circRNA research is concerned, although the role of circRNA in tumors has not been fully elucidated, there have been quite a few reports indicating that circRNA can be used as a competitive endogenous RNA to regulate tumors [48–50]. In this research, we confirmed that circLMO7 affected the WNT2/β-Catenin pathway by acting as a miR-30a-3p sponge to promote the development of GC. This is the first report that the ring structure of LMO7 can promote GC. However, this study only detected the expression of circLMO7 in GC tissues. Studies on the presence of circulating circLMO7 in patient blood and its impact on GC development have not been performed. This needs to be further studied in the future. To investigate the potential clinical significance of circLMO7, we investigated the relationship between circLMO7 and patient clinicopathological characteristics. We found that circLMO7 was closely related to the T grade and TNM stage of GC, indicating that circLMO7 can affect the infiltration and malignancy of GC. However, circLMO7 had no significant correlation with lymphatic invasion, which was inconsistent with our results in GC cells. We suspect that the migration and invasion capability were enhanced in GC cells, but this may not be specific to lymph node metastases. Metastases are the result of complicated molecular networks, and circLMO7 may contribute to only part of the process. Further research needs to be conducted to confirm this prediction. In the process of finding the downstream miRNA of circLMO7, we identified 11 potential miRNAs, of which miR-30a-3p had the highest binding efficiency, so we focused our study on this miRNA. We found that miR-30a-3p was downregulated in GC and inhibited the development of GC, which was consistent with other studies on miR-30a-3p [51–53]. However, whether circLMO7 can affect the development of GC through other miRNAs remains to be studied.

Glutamine metabolism is one of the important metabolic pathways in the development of cancer [54, 55]. It has been reported that circRNAs can promote the development of cancer through glutamine metabolism [21, 22], and the WNT2/β-Catenin signaling pathway is related to glutamine metabolism in cancer [35, 36]. Therefore, we confirmed the relationship between circLMO7 and glutamine metabolism in GC. In this study, circLMO7 mainly promoted the development of GC by regulating the activity of GLS to promote the TCA cycle and ROS stress balance. However, in recent years, some studies have reported that glutamine can be used as a nitrogen source to participate in the synthesis of nucleotides (purines and pyrimidines) [56, 57]. Moreover, glutamine is related to mTOR signaling, apoptosis, and autophagy [58–60]. These mechanisms provide new directions for our future research.

## Conclusions

Taken together, we have demonstrated that circLMO7 is significantly upregulated in GC tissues and that circLMO7 can act as a miR-30a-3p sponge affecting the WNT2/β-Catenin pathway to promote the proliferation, migration and invasion of GC cells. CircLMO7 can also promote the malignant biological functions of GC by facilitating glutaminolysis. In addition, HNRNPL can promote the self-cyclization of circLMO7. In conclusion, circLMO7 is expected to become a new biomarker for GC and a potential target for treatment (Supplementary Fig. 5).

## Supplementary Information

**Additional file 1: Fig. S1.** Validation of the circLMO7 circular structure. (A). Sanger sequencing revealed that circLMO7 originated from the LMO7 gene on human chromosome 13 and was circularized by exons 3, 4, and 5. (B). RNA fluorescence in situ hybridization (FISH) successfully localized circLMO7 in the cytoplasm; scale bar = 10 μm. (C). RNase R treatment suggested that circLMO7 was less susceptible to RNase R digestion than linear LMO7. (D). Agarose gel electrophoresis suggested that circLMO7 was more resistant to RNase R. (E). The actinomycin D inhibition test showed that the half-life of circLMO7 was significantly higher than that of linear LMO7. All data are presented as the mean ± SD. * P < 0.05, ** P < 0.01, *** *P* < 0.001.

**Additional file 2: Fig. S2.** The transfection efficiency of oligonucleotide sequences. (A). Transfection efficiency of ov-circLMO7. (B). Transfection efficiency of si-circLMO7. (C). Transfection efficiency of in-miR-30a-3p and mi-miR-30a-3p. (D). Transfection efficiency of si-GLS. (E). Transfection efficiency of si-HNRNPL. All data are presented as the mean ± SD. * P < 0.05, ** P < 0.01, *** P < 0.001.

**Additional file 3: Fig. S3.** CircLMO7 promotes the development of GC through the circLMO7-miR-30a-3p-WNT2 axis. (A, B). Colony formation and EdU assays showed that the inhibitory effect of si-circLMO7 on GC cell proliferation was rescued after cotransfection with in-miR-30a-3p; EdU scale bar = 25 μm. (C). Scratch assays showed that the inhibitory effect of si-circLMO7 on GC cell migration was rescued after cotransfection with in-miR-30a-3p; scale bar = 300 μm. (D). Transwell assays showed that the inhibitory effect of si-circLMO7 on GC cell migration and invasion was rescued after cotransfection with in-miR-30a-3p; scale bar = 100 μm. (E). Human GC organoid experiments showed that the inhibitory effect of si-circLMO7 on GC cell proliferation was rescued after cotransfection with in-miR-30a-3p; scale = 20 μm. (F). Western blot analysis showed that the activity of the EMT pathway and the expression levels of WNT2 and its downstream proteins were decreased when we transfected si-circLMO7. However, all of these effects were rescued after cotransfection with in-miR-30a-3p. All data are presented as the mean ± SD. * *P* < 0.05, ** *P* < 0.01, *** *P* < 0.001.

**Additional file 4: Fig. S4.** CircRNA-miRNA-mRNA pathway analysis suggested that the circLMO7-miR-30a-3p-WNT2 axis was closely related to glutamine metabolism.

**Additional file 5: Fig. S5** CircLMO7 mechanism diagram.

**Additional file 6: Table. 1** The expression of circLMO7 was closely related to the T grade and stage of GC.

**Additional file 7.**

## Data Availability

The datasets used and/or analyzed during the current study are available from the corresponding author on reasonable request.

## Abbreviations

GC: Gastric cancer
ceRNA: Competitive endogenous RNA
circRNA: Circular RNA
miRNA: MicroRNA
pre-mRNA: Precursor mRNA
GLS: Glutaminase
GLN: Glutamine
GLU: Glutamate
α-KG: α-ketoglutarate
GDH: Glutamate dehydrogenase
GSH: Glutathione
TCA cycle: Tricarboxylic acid cycle
ROS: Reactive oxygen species
RBP: RNA-binding protein
HNRNPL: Heterogeneous ribonucleoprotein L
NGS: Next-generation sequencing
qRT-PCR: Quantitative real-time polymerase chain reaction
FISH: RNA fluorescence in situ hybridization
EdU: 5-Ethynyl-2′-deoxyuridine
IHC: Immunohistochemistry
HE: Hematoxylin-eosin
IF: Immunofluorescence
RIP: RNA immunoprecipitation
gDNA: Genomic DNA
EMT: Epithelial-mesenchymal transition
