# Supplementary figures and images for: Circular RNA circLMO7 acts as a microRNA-30a-3p sponge to promote gastric cancer progression via the WNT2/β-catenin pathway
Source: J Exp Clin Cancer Res. 2021 Jan 5;40:6. doi: 10.1186/s13046-020-01791-9 (PMC7784001; doi:10.1186/s13046-020-01791-9)

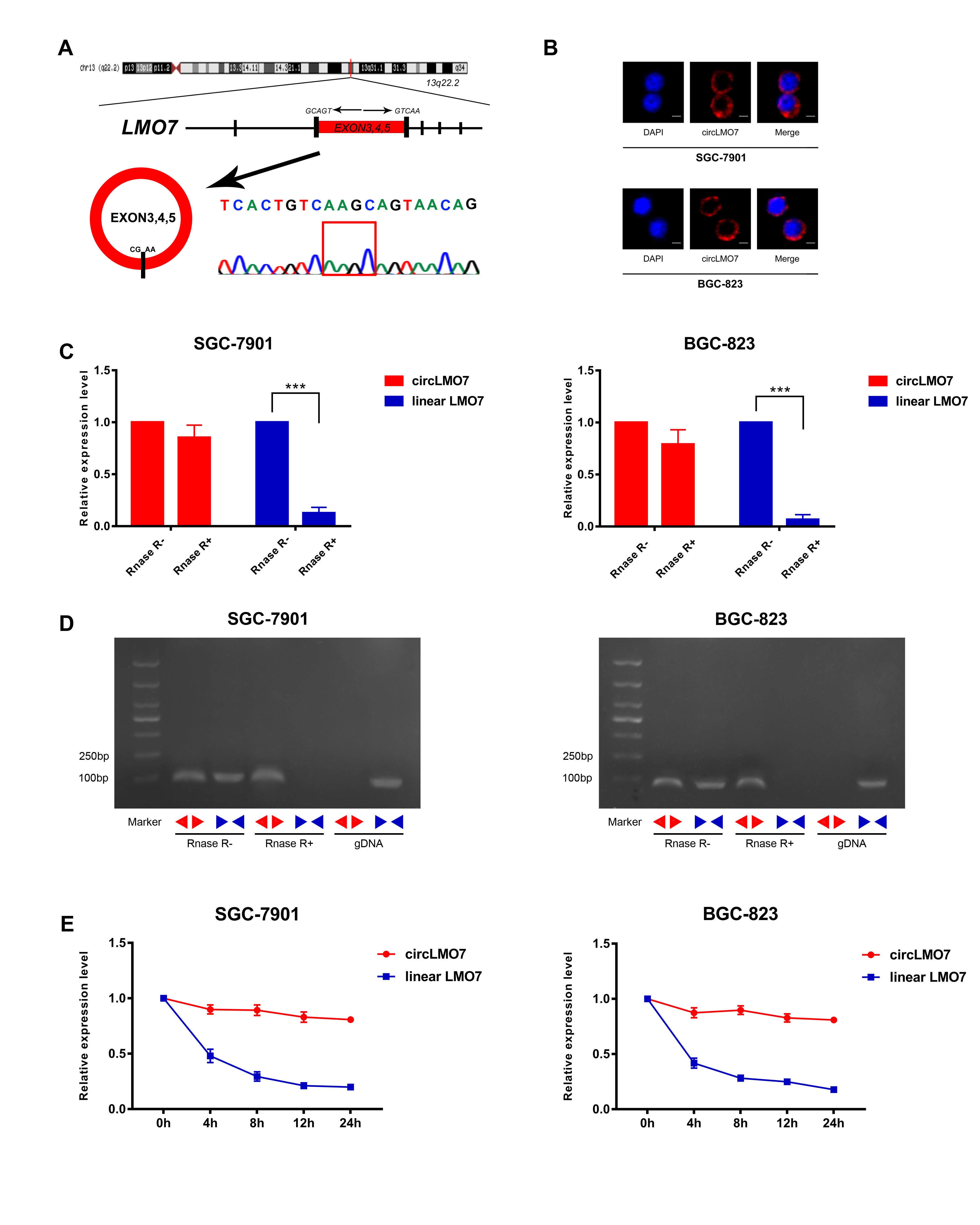

Supplement: Supplementary file 1 — Additional file 1: Fig. S1. Validation of the circLMO7 circular structure. (A). Sanger sequencing revealed that circLMO7 originated from the LMO7 gene on human chromosome 13 and was circularized by exons 3, 4, and 5. (B). RNA fluorescence in situ hybridization (FISH) successfully localized circLMO7 in the cytoplasm; scale bar = 10 μm. (C). RNase R treatment suggested that circLMO7 was less susceptible to RNase R digestion than linear LMO7. (D). Agarose gel electrophoresis suggested that circLMO7 was more resistant to RNase R. (E). The actinomycin D inhibition test showed that the half-life of circLMO7 was significantly higher than that of linear LMO7. All data are presented as the mean ± SD. * P < 0.05, ** P < 0.01, *** P < 0.001. [file 13046_2020_1791_MOESM1_ESM.tif]

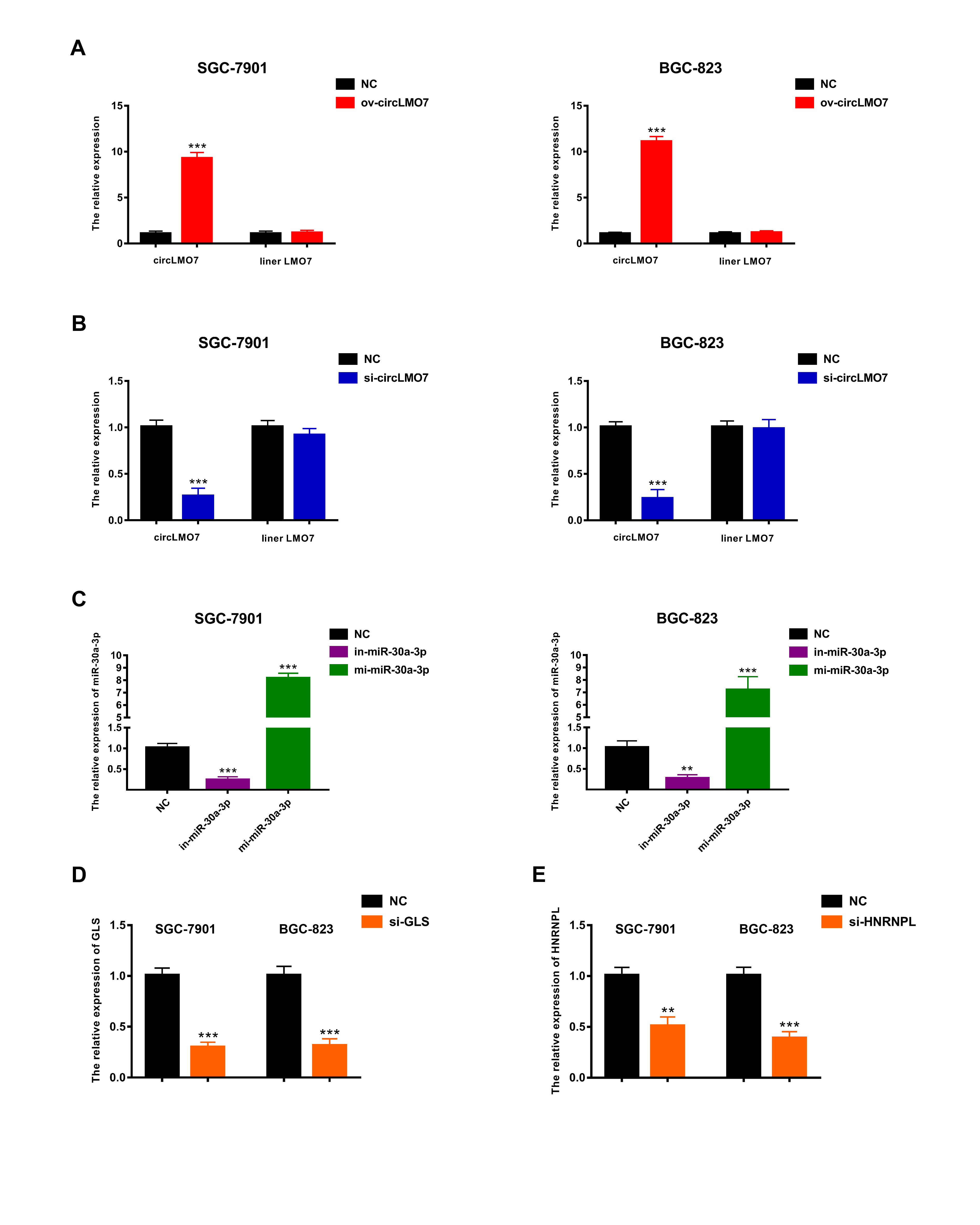

Supplement: Supplementary file 2 — Additional file 2: Fig. S2. The transfection efficiency of oligonucleotide sequences. (A). Transfection efficiency of ov-circLMO7. (B). Transfection efficiency of si-circLMO7. (C). Transfection efficiency of in-miR-30a-3p and mi-miR-30a-3p. (D). Transfection efficiency of si-GLS. (E). Transfection efficiency of si-HNRNPL. All data are presented as the mean ± SD. * P < 0.05, ** P < 0.01, *** P < 0.001. [file 13046_2020_1791_MOESM2_ESM.tif]

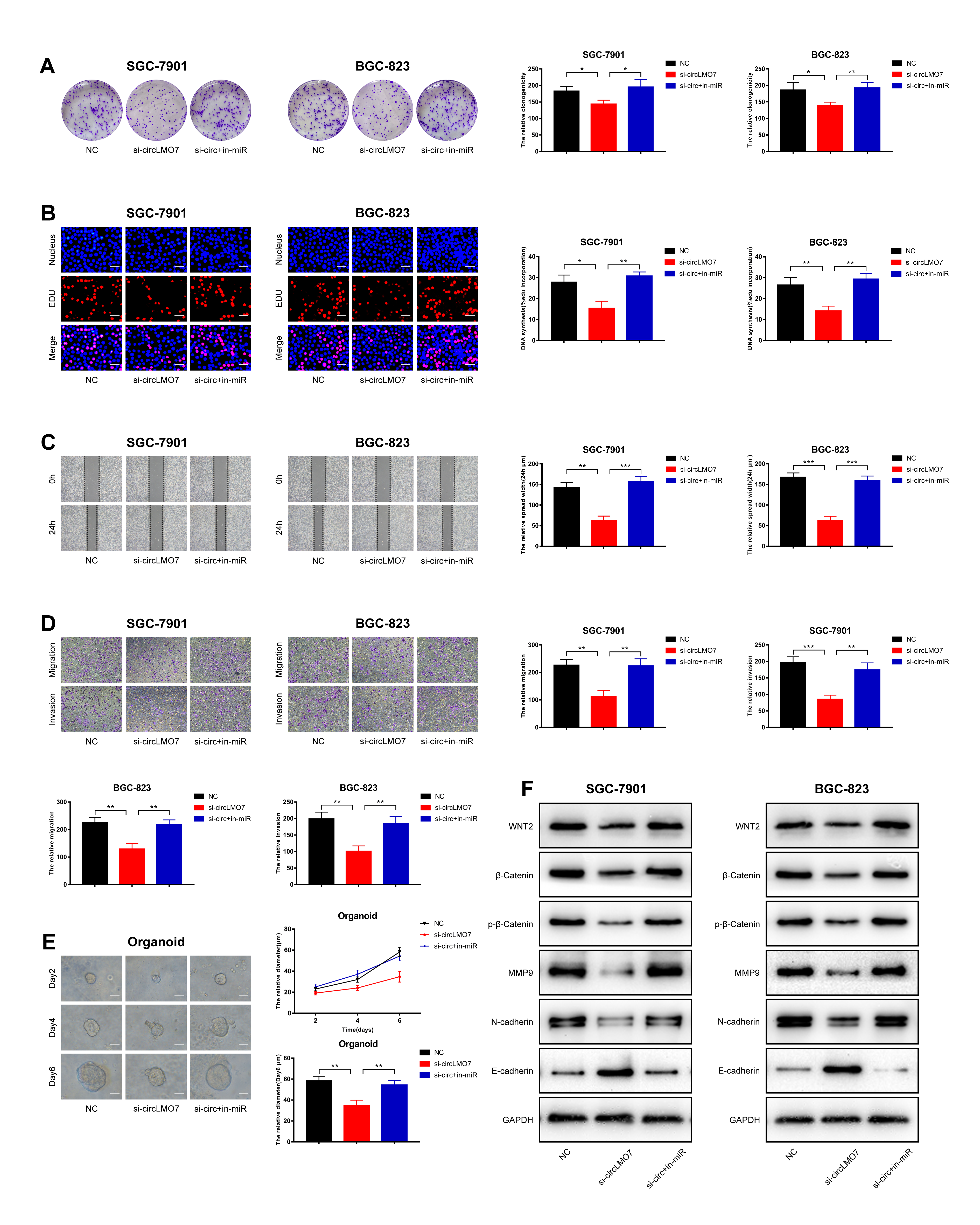

Supplement: Supplementary file 3 — Additional file 3: Fig. S3. CircLMO7 promotes the development of GC through the circLMO7-miR-30a-3p-WNT2 axis. (A, B). Colony formation and EdU assays showed that the inhibitory effect of si-circLMO7 on GC cell proliferation was rescued after cotransfection with in-miR-30a-3p; EdU scale bar = 25 μm. (C). Scratch assays showed that the inhibitory effect of si-circLMO7 on GC cell migration was rescued after cotransfection with in-miR-30a-3p; scale bar = 300 μm. (D). Transwell assays showed that the inhibitory effect of si-circLMO7 on GC cell migration and invasion was rescued after cotransfection with in-miR-30a-3p; scale bar = 100 μm. (E). Human GC organoid experiments showed that the inhibitory effect of si-circLMO7 on GC cell proliferation was rescued after cotransfection with in-miR-30a-3p; scale = 20 μm. (F). Western blot analysis showed that the activity of the EMT pathway and the expression levels of WNT2 and its downstream proteins were decreased when we transfected si-circLMO7. However, all of these effects were rescued after cotransfection with in-miR-30a-3p. All data are presented as the mean ± SD. * P < 0.05, ** P < 0.01, *** P < 0.001. [file 13046_2020_1791_MOESM3_ESM.tif]

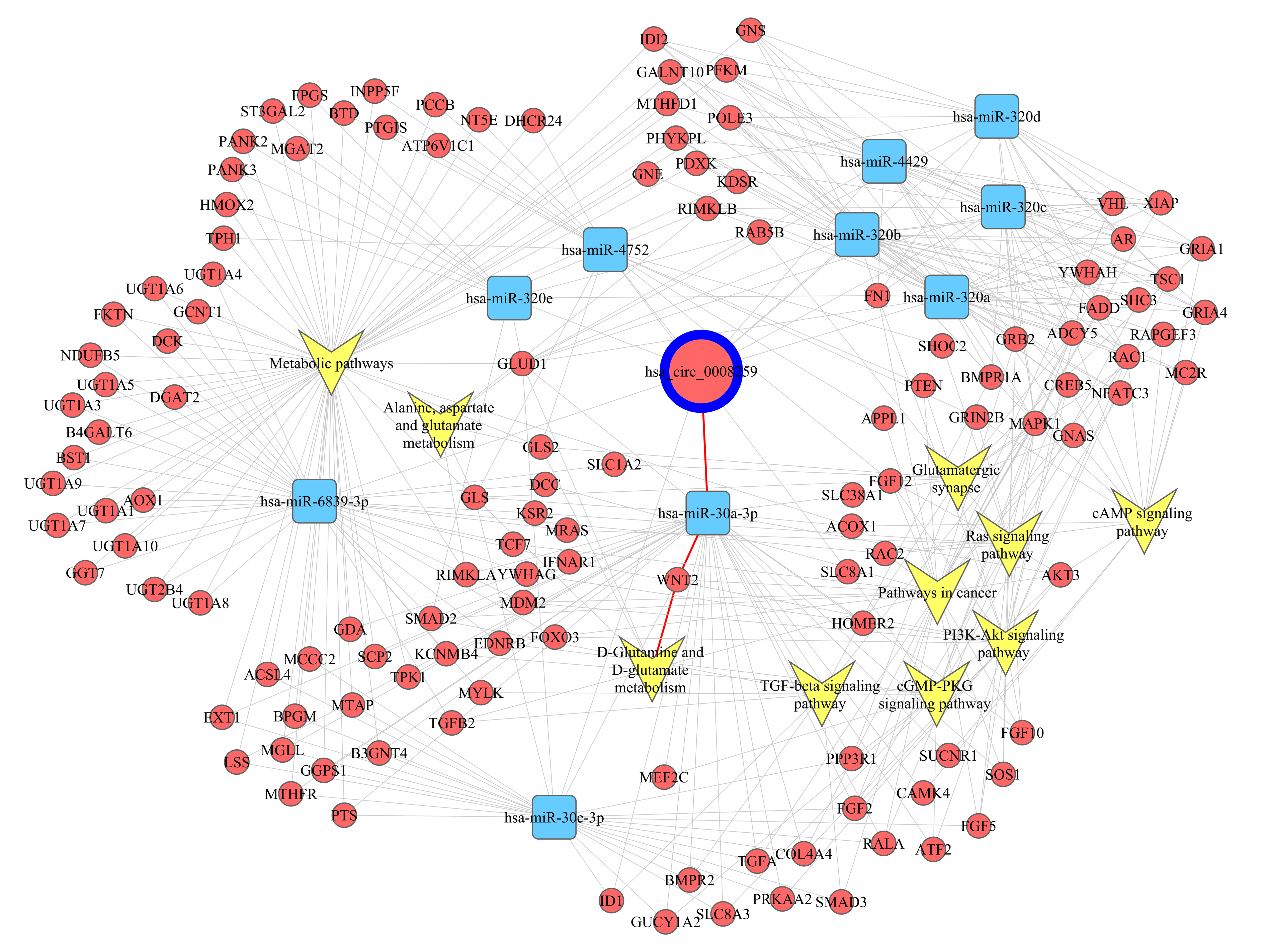

Supplement: Supplementary file 4 — Additional file 4: Fig. S4. CircRNA-miRNA-mRNA pathway analysis suggested that the circLMO7-miR-30a-3p-WNT2 axis was closely related to glutamine metabolism. [file 13046_2020_1791_MOESM4_ESM.tif]

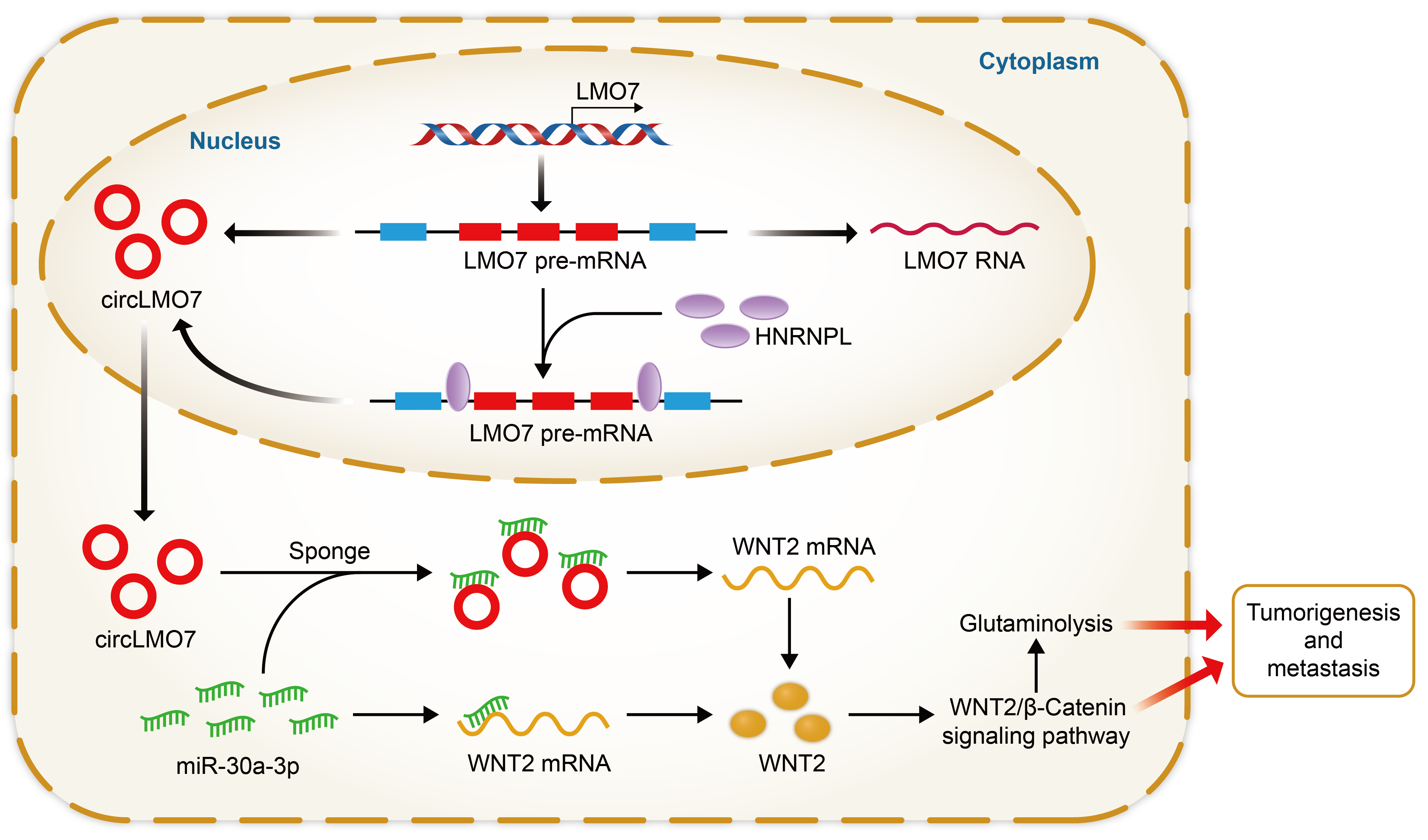

Supplement: Supplementary file 5 — Additional file 5: Fig. S5 CircLMO7 mechanism diagram. [file 13046_2020_1791_MOESM5_ESM.tif]
