# Supplementary material for: Circular RNA circLMO7 acts as a microRNA-30a-3p sponge to promote gastric cancer progression via the WNT2/β-catenin pathway
Source: J Exp Clin Cancer Res. 2021 Jan 5;40:6. doi: 10.1186/s13046-020-01791-9 (PMC7784001; doi:10.1186/s13046-020-01791-9)
Supplement: Supplementary file 6 — Additional file 6: Table. 1 The expression of circLMO7 was closely related to the T grade and stage of GC. [file 13046_2020_1791_MOESM6_ESM.docx]

Supplementary Table 1

| Primer sequence |  |
| --- | --- |
| circLMO7 | Forward: 5'-CAAATCGAGTCACTGTCAAGCA-3' Reverse: 5'-TCACACAGCAGAACACCATTTT-3' |
| linear LMO7 | Forward: 5'-TCTTGAAAGCTTGTGAACAGATTGG-3' Reverse: 5'-CAGTGACTCGATTTGATAAATCCTG-3' |
| miR-320c | Forward: 5'-AAAAGCTGGGTTGAGAGGGT-3' Reverse: General downstream primer  5'-CAGTGCGTGTCGTGGAGT-3' |
| miR-103a-2-5p | Forward: 5'-AGCTTCTTTACAGTGCTGCCTTG-3' Reverse: General downstream primer' |
| miR-30e-3p | Forward: 5'-CTTTCAGTCGGATGTTTACAGC-3' Reverse: General downstream primer' |
| miR-21-3p | Forward: 5'-CAACACCAGTCGATGGGCTGT-3' Reverse: General downstream primer' |
| miR-320d | Forward: 5'-AAAAGCTGGGTTGAGAGGA-3' Reverse: General downstream primer' |
| miR-3688-3p | Forward: 5'-TATGGAAAGACTTTGCCACTCT-3' Reverse: General downstream primer |
| miR-3591-3p | Forward: 5'-AAACACCATTGTCACACTCCAC-3' Reverse: General downstream primer' |
| miR-30a-3p | Forward: 5'-CTTTCAGTCGGATGTTTGCAGC-3' Reverse: General downstream primer' |
| miR-320b | Forward: 5'-AAAAGCTGGGTTGAGAGGGCAA-3' Reverse: General downstream primer' |
| miR-320a | Forward: 5'-AAAAGCTGGGTTGAGAGGGCGA-3' Reverse: General downstream primer' |
| miR-4429 | Forward: 5'-AAAAGCTGGGCTGAGAGGCG-3' Reverse: General downstream primer' |
| WNT2 | Forward: 5'-AAGAAGATGGGAAGCGCCAA-3' Reverse: 5'-ACCGCTTTACAGCCTTCCTG-3' |
| GLS1 | Forward: 5'-TGCATTCCTGTGGCATGTAT-3' Reverse: 5'-TTGCCCATCTTATCCAGAGG-3' |
| HNRNPL | Forward: 5'-TTCTGCTTATATGGCAATGTGG-3' Reverse: 5'-GACTGACCAGGCATGATGG-3' |
| GAPDH | Forward: 5'-GAACGGGAAGCTCACTGG-3' Reverse: 5'-GCCTGCTTCACCACCTTCT-3' |
| U6 | Forward: 5'-CTCGCTTCGGCAGCACA-3' Reverse: General downstream primer |
| Transfection sequence |  |
| si-circLMO7 | 5'-CUUCUCUGUUACUGCUUGA-3' |
| miR-30a-3p mimics | 5'-CUUUCAGUCGGAUGUUUGCAGC-3' 5'-GCUGCAAACAUCCGACUGAAAG-3' |
| miR-30a-3p inhibitors | 5'-GCUGCAAACAUCCGACUGAAAG-3' |
| si-GLS | 5'-AUUUCGAACUGCUUCAGGG-3' |
| si-HNRNPL | 5'-GAAUGGAGUUCAGGCGAUG-3' |
| Fluorescent probe sequence |  |
| circLMO7 | 5'-Cy3-AAATTCTTCTCTGTTACTGC  TTGACAGTGACTCGATTTGA-Cy3-3' |
| miR-30a-3p | 5'-Fam-GCTGCAAACATCCGACTGAAAG-Fam–3' |
| Biotinylated probe sequence |  |
| circLMO7 | 5'-CTGTTACTGCTTGACAGTGA-bio-3' |
| Luciferase reporter gene mutation sequence |  |
| circLMO7(mut145-166) | 5'-AAAAUUUUGGAUUUCUACUUUC-3' |
| circLMO7(mut167-190) | 5'-UGACAUCUGUAUGCUUACUUUC-3' |
| WNT2(mut) | 5'-CAACGUUUGUCAUAUGACUUUC-3' |
| I2HB(mut) | 5'-UGUGUGU-3' 5'-UGUGUGU-3' |
| I5HB(mut) | 5'-UGUUUUU-3' 5'-UUUGUGU-3' |

Supplementary Table 2

| Primary antibody |  |  |
| --- | --- | --- |
| WNT2 | Abcam | ab27794 |
| β-catenin | Cell signaling Technology | #8480 |
| p-β-catenin | Cell signaling Technology | #1187 |
| MMP9 | Cell signaling Technology | #13667 |
| N-cadherin | Cell signaling Technology | #13116 |
| E-cadherin | Cell signaling Technology | #14472 |
| GAPDH | Cell signaling Technology | #5174 |
| GLS1 | Cell signaling Technology | #56750 |
| HNRNPL | Abcam | ab6106 |
| Secondary antibody |  |  |
| Anti-rabbit IgG | Cell signaling Technology | #7074 |
| Anti-mouse IgG | Cell signaling Technology | #7076 |
