# Supplementary material for: Circular RNA circLMO7 acts as a microRNA-30a-3p sponge to promote gastric cancer progression via the WNT2/β-catenin pathway
Source: J Exp Clin Cancer Res. 2021 Jan 5;40:6. doi: 10.1186/s13046-020-01791-9 (PMC7784001; doi:10.1186/s13046-020-01791-9)
Supplement: Supplementary file 7 — Additional file 7. [file 13046_2020_1791_MOESM7_ESM.docx]

**Supplementary Materials and Methods**

**Clinical tissue specimens**

Clinical tissue specimens used for qRT-PCR and immunohistochemistry were obtained from the First Affiliated Hospital of Nanjing Medical University in China. The time span of these tissue specimens was from 2015 to 2019. After surgery, all tissue specimens were quickly placed into liquid nitrogen for storage. The pathological diagnosis of the tissue specimens was independently performed by two pathologists. None of the patients in this study received any treatment related to gastric cancer before surgery. All research was carried out with the informed consent of patients.

**Next-generation sequencing analysis**

We collected three pairs of GC tissue samples and their corresponding adjacent tissues for next-generation sequencing (NGS) analysis. We used TRIzol reagent (Invitrogen, Carlsbad, CA, USA) to extract RNA and reverse transcribed the RNA into cDNA by using random primers. Then, we synthesized the second-strand cDNA and used magnetic beads to purify the ligated double-stranded cDNA. After that, we used uracil DNA glycosylase to remove the second-strand cDNA and amplified the remaining cDNA by PCR. Finally, we used HiSeq 2500 (Illumina, USA) to test the RNA-seq libraries.

**RNA extraction and qRT-PCR**

According to the manufacturer's instructions, total RNA in tissues/cells was extracted by TRIzol reagent (Invitrogen, Carlsbad, CA, USA). The amount and integrity of the RNA were detected by a Nanodrop 2000 (Thermo Fisher Scientific, Waltham, MA, USA). The PrimeScript ™ RT Master Mix Kit (RR036A, Takara, Japan) was used to reverse transcribe RNA into cDNA. We used the FastStart Universal SYBR Green Master Kit (Roche, Mannheim, Germany) for quantitative real-time polymerase chain reaction (qRT-PCR). This process was performed using the ABI PRISM 7900HT Sequence Detection System (Applied Biosystems, Waltham, MA, USA). The primer sequences used in qRT-PCR are shown in Supplementary Table 1.

**Cell culture**

The human cell lines (GES-1, HGC-27, SGC-7901, MGC-803, BGC-823, MKN-45) used in this research were purchased from the Shanghai Institute of Biological Sciences. These cells were cultured in RPMI 1640 (Gibco, Carlsbad, CA, USA) containing 1% penicillin-streptomycin (Gibco, Carlsbad, CA, USA) and 10% fetal bovine serum (Gibco, Carlsbad, CA, USA). All of the cells were incubated in a 5% CO_2_ incubator at 37°C.

**RNA fluorescence in situ hybridization (FISH)**

The fluorescent probes of circLMO7 and miR-30a-3p were synthesized by Servicebio (Wuhan, China). Their sequences are shown in Supplementary Table 1. During the experiment, we seeded cells on glass slides and fixed them with 4% paraformaldehyde. After permeabilization with 0.25% Triton X-100 and washing with sodium citrate buffer (SSC), we incubated the cells with DIG-labeled probes in a hybridization buffer at 37°C overnight. Next, we washed and mixed the reaction system with SSC and anti-DIG-HRP (PerkinElmer, NEF832001EA, USA), respectively, and incubated it at 4°C overnight. The next day, the reaction system was washed again and incubated with TSA fluorescent signal reaction solution (PerkinElmer, NEL701001KT, USA) at room temperature for 30 minutes. After that, the DNA was stained with 4',6-diamidino 2-phenylindole (DAPI), and the cells were observed under a Leica SP5 confocal microscope (Leica Microsystems, Wetzlar, Germany).

**RNase R treatment**

A total of 3 U/μg RNase R (Epicentre Technologies, Madison, WI, USA) was added to total RNA (2 μg) and incubated at 37°C for 30 minutes. Reverse transcription was then performed on the treated RNA. The stability of circLMO7 was tested by qRT-PCR.

**Actinomycin D treatment**

Actinomycin D (2 mg/ml, Sigma-Aldrich, St. Louis, MO, USA) was added to the cells and cultured at 37°C. Then, we extracted RNA from cells at different times (0 h, 4 h, 8 h, 12 h, 24 h) and performed reverse transcription. The stability of circLMO7 was tested by qRT-PCR.

**Transfection of small interfering RNAs, plasmids, lentiviruses, miRNA mimics and inhibitors**

The oligonucleotide sequences used for transfection were constructed by GenePharma (Shanghai, China). The reagents included small interfering RNA against circLMO7 (si-circLMO7), the overexpression plasmid of circLMO7 (ov-circLMO7), the knockdown sequence of circLMO7 expressed by a lentivirus (ANTI-circLMO7), the overexpression sequence of circLMO7 expressed by a lentivirus (LV-circLMO7), miR-30a-3p mimics (mi-miR-30a-3p), and miR-30a-3p inhibitor (in-miR-30a-3p). The specific sequences are shown in Supplementary Table 1. The transfection reagent was Lipofectamine 3000 (Thermo Fisher Scientific, Waltham, MA, USA), and the transfection procedure was performed according to the manufacturer's instructions.

**Colony formation assay**

Cells were seeded into six-well plates at a density of 1x10^3^ cells per well. After 14 days of incubation, the cells were fixed with 75% alcohol and stained with 0.1% crystal violet. After that, the cell colonies were observed and counted.

**5-Ethynyl-2’-deoxyuridine (EdU)**

The reagents required for EdU were derived from the Cell-Light ^TM^ EdU DNA Cell Proliferation Kit (RiboBio, Guangzhou, China). The experiment was performed as follows. First, each well of the 96-well plate was seeded with 1×10^4^ cells. The next day, 50 μM EdU medium was added to each well for 2 h After that, the cells were fixed with 4% paraformaldehyde and stained with Appllo. DNA was stained with Hoechst 33342, and images were captured with an Olympus microscope (Olympus, Tokyo, Japan).

**Transwell assay**

We placed Transwell inserts (Millipore, Billerica, MA, USA) in a 24-well plate. Then, the cells were resuspended in serum-free medium. The upper chamber of the Transwell insert was inoculated with 200 μl resuspension to ensure that there were 3×10^4^ cells per well. A total of 600 μl complete medium containing 10% FBS was directly added to the lower chamber of the Transwell insert. After 24 h, the inside of the filter was wiped with a cotton swab, and the filter was stained with 0.1% crystal violet. The cell morphology was observed under a microscope. To verify cell invasion, we applied 100 μl of Matrigel (BD Bioscience, San Jose, CA, USA) to the inside of the filter before inoculating cells in the upper chamber. The remaining steps were the same as above.

**Wound-healing assay**

We seeded the cells into a six-well plate at a density of 3x10^5^ cells per well. When the cell confluence reached 90%, a 200 μl sterile pipette tip was used to scratch the center of each well. After that, we washed the cells with PBS and observed them under a microscope.

**Human organoid culture**

Minced human cancer tissue was digested by collagenase A at 37°C for 30 minutes. During this period, the system was oscillated every 10 minutes. Next, the digested product was filtered with a strainer and centrifuged at 4°C to purify cancer cells. At the same time, growth factors and antibiotics were added to Matrigel (R & D Systems, Minneapolis, MN, USA) and Organoid Growth Medium (human) (StemCell Technologies, Vancouver, Canada). After that, cells were resuspended in 50 μl prepared Matrigel and seeded on a 24-well plate. Then, 500 μl of prepared medium was added to each well and cultured in an incubator (37°C, 5% CO_2_). Transfection of oligonucleotide sequences was performed by Lipofectamine 3000. The growth of organoids was observed under a microscope every 2 days.

**Protein extraction and Western blot**

RIPA lysis buffer (Servicebio, Wuhan, China) and protease and phosphatase inhibitor cocktail (NCM Biotech, Suzhou, China) were added to the cells to extract the protein. Then, we added SDS-PAGE Protein Buffer (Beyotime, Shanghai, China) to the protein sample and heated it in boiling water for 3-5 minutes to denature the protein. The protein was added to 10% PAGE gel (Epizyme, Shanghai, China) at an amount of 10 μl/well and electrophoresed in SDS-PAGE Running Buffer (Servicebio, Wuhan, China). Next, we transferred the protein to a PVDF membrane (Thermo Fisher Scientific, Waltham, MA, USA) by SDS-PAGE Transfer Buffer (Servicebio, Wuhan, China). After blocking with Quickblock ^TM^ Buffer (Beyotime, Shanghai, China), the protein was incubated with the primary antibody at 4°C overnight. The next day, the protein was incubated with the secondary antibody for 2 h. After that, we added chemiluminescence HRP substrate (Millipore, Billerica, MA, USA) to the protein and used the BioSpectrum 600 Imaging System (Thermo Fisher Scientific, Waltham, MA, USA) to obtain immunoblot images. Information on the antibodies is shown in Supplementary Table 2.

**Immunohistochemistry (IHC) analysis and hematoxylin-eosin (HE) staining**

We cut the tissues into paraffin sections at a thickness of 5 μm. After dewaxing and blocking, we incubated the paraffin sections with the primary antibody at 4°C overnight. The next day, we incubated the sections with the secondary antibody for 1 h. Next, we used the DAB Staining Kit (Servicebio, Wuhan, China) and hematoxylin (Beyotime, Shanghai, China) to stain the protein and nucleus, respectively. The HE staining procedure was slightly different from the IHC procedure. The nucleus and cytoplasm were directly stained with a hematoxylin-eosin staining kit (Beyotime, Shanghai, China) after dewaxing. Information on the antibodies is shown in Supplementary Table 2.

**Immunofluorescence (IF) analysis**

The reagents required for IF were obtained from Beyotime (Shanghai, China). On the first day, cells were seeded on confocal dishes (Solarbio, Beijing, China) at 3×10^4^ cells per well. The next day, the cells were sequentially treated with fixative solution and permeabilization solution and then incubated with the blocking solution for 2 h. After that, we incubated the cells with the primary antibody at 4°C overnight. On the third day, we incubated the cells with the secondary antibody for 1 h. The nuclei were stained with a DAPI Staining Kit, and the cells were observed under an LSM 710 confocal microscope (Zeiss, Germany). Information on the antibodies is shown in Supplementary Table 2.

**Animal experiments**

The 4-week-old BALB/c nude mice used in the experiment were purchased from the Experimental Animal Center of Nanjing Medical University. Before the experiments, we transfected the lentiviral-circularized circLMO7 knockdown sequence (ANTI-circLMO7), the lentiviral-circularized circLMO7 overexpression sequence (LV-circLMO7), and the negative control sequence into cells by Lipofectamine 3000. For the xenograft tumor models, we injected the cells into the forelimb underarms of each mouse at an amount of 1×10^6^, and the volume of xenograft tumors was measured once a week (V=length×width^2^×0.5). All nude mice were sacrificed after 4 weeks, and the weight of xenograft tumors was measured. For the lung metastasis models, we injected the cells into the tail vein of each mouse at an amount of 1×10^6^. Four weeks later, with the help of D-Luciferin (Caliper Life Sciences, Waltham, MA USA), lung metastasis was observed by the IVIS imaging system 200 series (Xenogen Corporation, Waltham, MA, USA).

**The detection of glutamine, glutamate and α-KG**

We used a Glutamine/Glutamate Assay Kit (Sigma-Aldrich, St. Louis, MO, USA) to measure Glutamine, Glutamate levels. α-KG lever was measured by α-KG Assay Kit (Abcam, Cambridge, MA, USA). All the measurements are carried out according to the manufacturer’s instructions.

**Reactive oxygen species (ROS) assay**

We seeded the cells on confocal dishes (Solarbio, Beijing, China) at an amount of 3×10^4^ cells per well. The next day, DCFH-DA was added to the cells according to the instructions of the ROS Assay Kit (Beyotime, Shanghai, China). The treated cells were incubated for 30 minutes and observed under an LSM 710 confocal microscope (Zeiss, Germany).

**RNA pull-down assay**

We mixed the biotinylated circLMO7 probe (RiboBio, Guangzhou, China) and the C-1 magnetic beads (Life Technologies, Waltham, MA, USA) at room temperature for 20 minutes. Next, the magnetic beads were collected and resuspended with the cell lysate prepared in advance. The system was slowly rotated at 4°C for 2 h. The magnetic beads were collected again, and the RNA complex bound to the beads was extracted. qRT-PCR was used to verify the expression of relevant specific miRNAs in the RNA complex. Information on the circLMO7 probe is shown in Supplementary Table 1.

**Luciferase reporter assay**

We seeded cells on a 96-well plate. When the cell confluence reached 70%, the Firefly Luciferase Reporter Vector (GenePharma, Shanghai, China) containing circLMO7 3'UTR, the Renilla Luciferase Reporter Vector (GenePharma, Shanghai, China) and miR-30a-3p mimics were transfected into cells by Lipofectamine 3000. After 48 h, the luciferase activity was measured by the Dual-Luciferase Reporter Assay System (Promega, Madison, WI, USA). In this experiment, we verified the binding site between circLMO7 and miR-30a-3p by mutating the circLMO7 3'UTR. This assay can also be used to verify the binding site between miRNA and mRNA. Related mutation sequences are shown in Supplementary Table 1.

**RNA** **immunoprecipitation (RIP) assay**

A Magna RIP RNA Binding Protein Immunoprecipitation Kit (Millipore, MA, USA) was used to perform the RIP assay. We added RBP-specific antibodies to the pretreated protein lysate and incubated them at 4°C overnight. The next day, we added magnetic beads to the above system and incubated them at 4°C. 2 h later, we eluted the beads and extracted the RNA complex for qRT-PCR. The antibody information is shown in Supplementary Table 2.

**Statistical analysis**

SPSS 20.0 (IBM, SPSS, Chicago, IL, USA) and GraphPad Prism 7.0 (GraphPad Software, La Jolla, CA, USA) were applied for statistical analyses. In functional assays, we used Student's t test to analyze the data of 2 groups. Analysis of variance (ANOVA) was used to analyze the data of multiple groups. In addition, we used the chi-square test and Fisher's exact test to analyze the relationship between circLMO7 and clinicopathological characteristics. The Pearson correlation coefficient was used to analyze the correlation between the two variables as well. The data in this article are expressed as the mean ± SD, and P<0.05 was considered statistically significant.
